# Characterization of silk-like materials from larval salivary glands of the dipteran, *Keroplatus testaceus*

**DOI:** 10.1038/s41467-026-78291-5

**Published:** 2026-10-05

**Authors:** Peter Kmet, Jakub Opelka, Thayna Bisson Ferraz Lopes, Hana Sehadova, Ivo Sauman, Miluse Hradilova, Martin Humenik, Michal Zurovec

**Affiliations:** 1 Biology Centre CAS, Institute of Entomology, Branisovska 31, 370 05 Ceske Budejovice, Czech Republic; 2 University of South Bohemia, Faculty of Science, Branisovska 31, 370 05 Ceske Budejovice, Czech Republic; 3 State University of Londrina, Department of General Biology, Rodovia Celso Garcia Cid, PR 445 Km 380, Campus Universitário, P.O. Box 10,011, 86057-970 Londrina, Parana Brazil; 4 Institute of Molecular Genetics of the Czech Academy of Sciences, Videnska 1083, 142 00 Prague, Czech Republic; 5 University of Bayreuth, Department of Biomaterials, Prof.-Rüdiger-Bormann-Str. 1, 95447 Bayreuth, Germany

**Keywords:** Proteomics, Proteome, Entomology

## Abstract

Silk-like secretions are well-studied in many insects but remain poorly characterized in Diptera. Larvae of the dipteran *Keroplatus testaceus* produce two distinct silk-like materials—a mucus web and a cocoon—from their labial salivary glands, differing in composition and function. Here we combined histology, transcriptomics, electron microscopy, proteomics, mechanical testing, and FTIR spectroscopy to characterize these secretions. Neither contains fibroin; instead, both comprise hydrophilic proteins including collagen-like, mucin, and novel proline-rich proteins. Structural predictions and FTIR analyses reveal β-sheets, α-helices, and polyproline II helices, contributing to mechanical robustness despite high hydration. The mucus web facilitates larval mobility and spore capture, while the cocoon provides mechanical protection during pupation. These findings reveal a fibroin-independent silk system adapted to humid, microbe-rich environments, expanding understanding of insect silk diversity and offering insights into novel biomaterials.

## Abstract (web summary)

Keroplatus testaceus uses a fibroin-free silk system composed of hydrophilic, collagen-like, and β-sheet proteins to produce two materials: a stretchable, adhesive mucus web for feeding and movement, and a stronger cocoon for pupal protection.

## Introduction

Natural silk fibers are protein-based materials known for their excellent biocompatibility, mechanical strength, and controllable biodegradability^1^. The best-known examples of silk are produced by spiders, lepidopteran caterpillars, and trichopteran larvae. Lepidopteran larvae secrete silk fibers composed primarily of fibroins, structural proteins that confer tensile strength and extensibility. In arthropod groups outside of Lepidoptera and Trichoptera, silk-like secretions appear to have evolved independently^2^, often reflecting the ecological demands of specific species. Consequently, the composition and structural organization of these silk proteins vary considerably. Although many insects, including wasps, bees, ants, fleas, fireflies, mosquitoes, and fungus gnats, produce silk-like materials, the underlying structural and functional properties of these secretions remain largely unknown.

Fungus gnats of the dipteran family Keroplatidae comprise nearly 100 genera and approximately 1,000 species distributed globally, most commonly found in association with wood-decaying fungi^3,4^. The larvae of these species secrete silk-like webs from their labial salivary glands, which serve different ecological functions depending on the species: prey capture in predatory taxa or spore collection in fungivorous ones^5–7^. The most widely studied member of this family is the bioluminescent predator *Arachnocampa luminosa*, which inhabits the humid caves of New Zealand. Its larvae produce fishing lines and nests composed of hydrophilic proteins^8–10^. In contrast, larvae of *Bradysia coprophila*, a soil-dwelling sciarid, feed on decaying organic matter, including fungi and live plant tissues such as roots, bulbs, and seedlings^11^. *B. coprophila* is a significant pest in greenhouse and nursery systems, with both larval and adult stages contributing to the spread of a wide range of plant pathogens^12^.

*K. testaceus* is a lesser-known keroplatid species that inhabits moist, fungus-rich wooded environments in Europe. Both the larvae and pupae are bioluminescent, with light emission arising from proteinaceous granules localized in the fat body^13^. Larvae secrete ephemeral mucus webs along fungal substrates and later produce a more durable, rigid cocoon for pupation^6^. The dual use of secretions from a single pair of salivary glands provides a rare opportunity to examine two distinct silk-like materials, mucus webs and cocoons, generated by the same organ.

Despite the widespread occurrence of silk-like secretions in dipteran larvae, the molecular composition, structural organization, and mechanical properties of these materials remain undefined. To address this gap, the aim of this study was to comprehensively characterize the mucus web and cocoon secretions of *K. testaceus* larvae using an integrated approach that combined optical and electron microscopy, proteomic analyses, transcriptome data, mechanical assays, and structural characterization via FTIR. Our results reveal that *K. testaceus* silk lacks canonical fibroin-like proteins and instead comprises proline-rich proteins and other specialized components suited to humid, microbe-rich environments. This unique silk system supports a range of larval functions, including adhesion, locomotion, spore collection, and pupal protection.

## Results

### *K. testaceus* produces two types of silk products

**Fig. 1:**
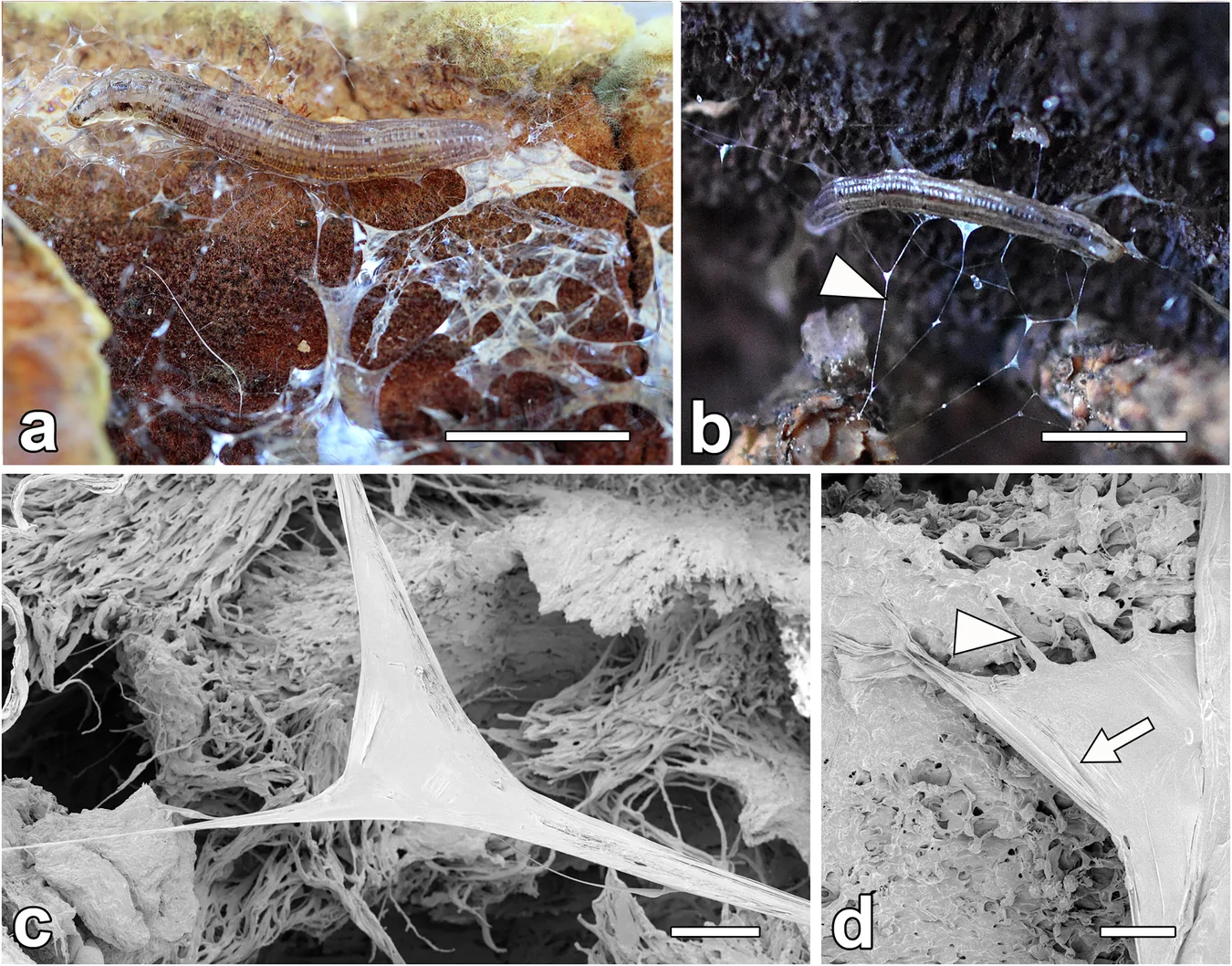
Macrophotographs and SEM views showing mucus production and structures formed by last-instar larvae *of K. testaceus*. **a, b** Larvae covered with a slimy coating, producing a mucus net composed of membranes and thin fibers (arrowhead). **c** SEM image showing a thin mucus membrane gradually transitioning into fine fibers. **d** Fibers (arrowhead) and membrane sheets (arrow) attached to the substrate. Scale bars: (**a, b**) 10 mm; (**c**) 40 μm; (**d**) 20 μm.

*K. testaceus* inhabits highly humid environments rich in wood-decaying fungi, which release abundant spores. Consequently, larvae live in an “upside-down world” on the pore surfaces of mature bracket fungi (e.g., *Fomitopsis pinicola* or *Fomes fomentarius*) growing on conifer stumps, or on the undersides of partially decayed conifer trunks. As shown in Fig. 1a, b, larvae are covered in a slimy coating and produce a mucus net composed of cohesive membranes and thin fibers. Flat, membrane-like structures within this net trap fungal spores, the larvae’s primary food source (Supplementary Fig. 1). These membranes also serve as tracks facilitating larval movement. The net is highly elastic when wet and can be drawn into fiber-like strands that act as attachment straps and safety lines, enabling smooth and secure movement across surfaces. This structural organization is evident in scanning electron microscopy (SEM) images (Fig. 1c), where a thin membrane extends above the surface and gradually transitions into fine fibers that connect to the substrate (Fig. 1d). These fibers establish stable anchoring points, offering mechanical support and fixation to the net during larval movement.

**Fig. 2:**
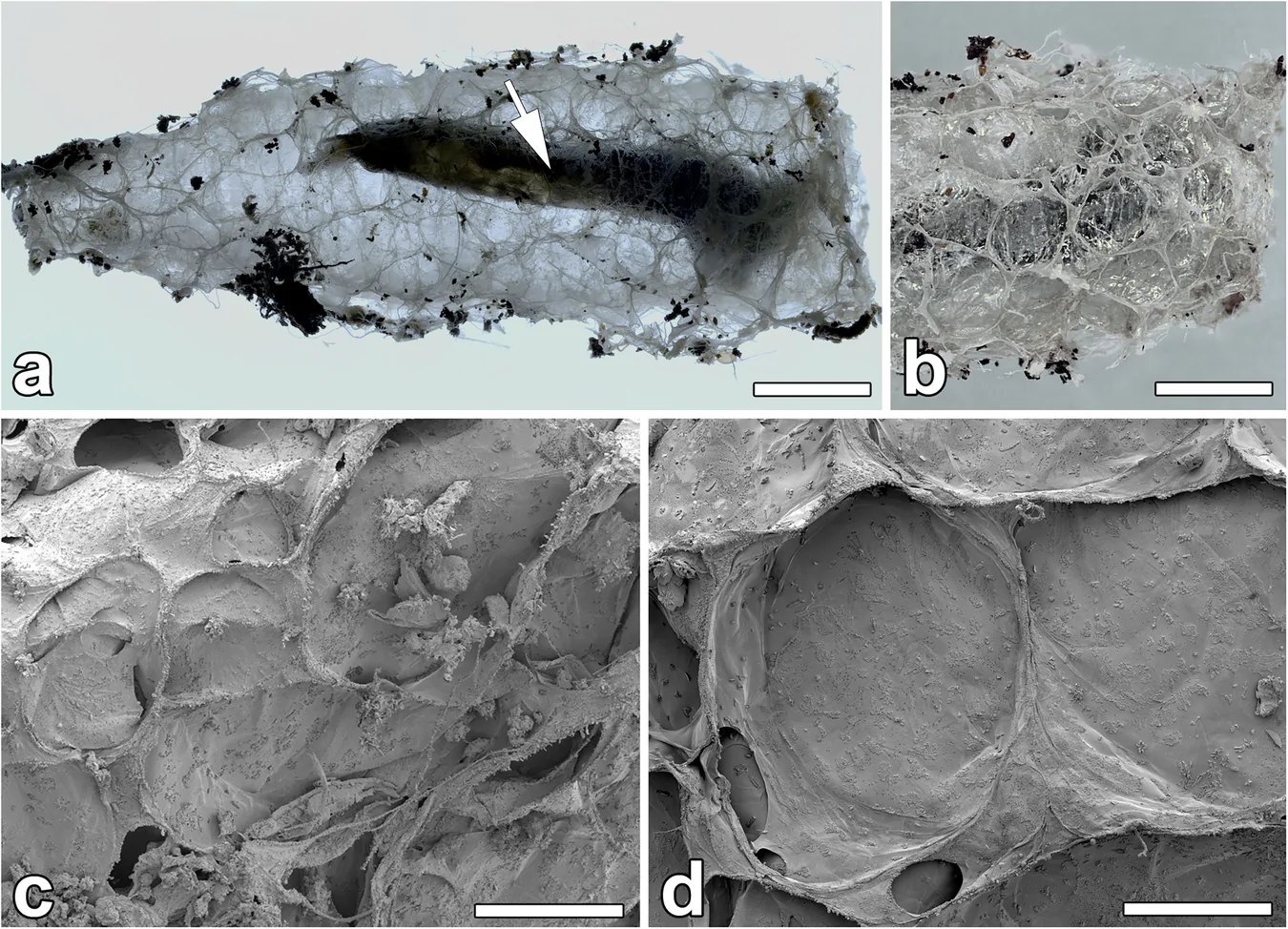
Morphology and microstructure of the *K. testaceus* cocoon. **a, b** Bright-field microscopy images of cocoons; arrow (**a**) points to the pupa within. **c, d** SEM micrographs of the cocoon wall showing a fibrous framework forming circular units on the outer surface. Scale bars: (**a, b**) 2 mm; (**c, d**) 500 μm.

In addition to mucus production, larvae construct cocoons before pupation. The cocoon is a compact, translucent structure supported by a framework of intertwined fibrils (Fig. 2a, b). Due to its transparency, the dark outline of the pupa is visible inside (Fig. 2a, arrow). SEM analysis confirmed that the cocoon consists of a network of interconnected fiber-like elements forming circular units, which are internally linked by a mucous layer (Fig. 2c). These internal connections are visible as raised ridges on the outer surface of the cocoon (Fig. 2d).

### Morphology of the salivary glands in *K. testaceus* larvae

**Fig. 3:**
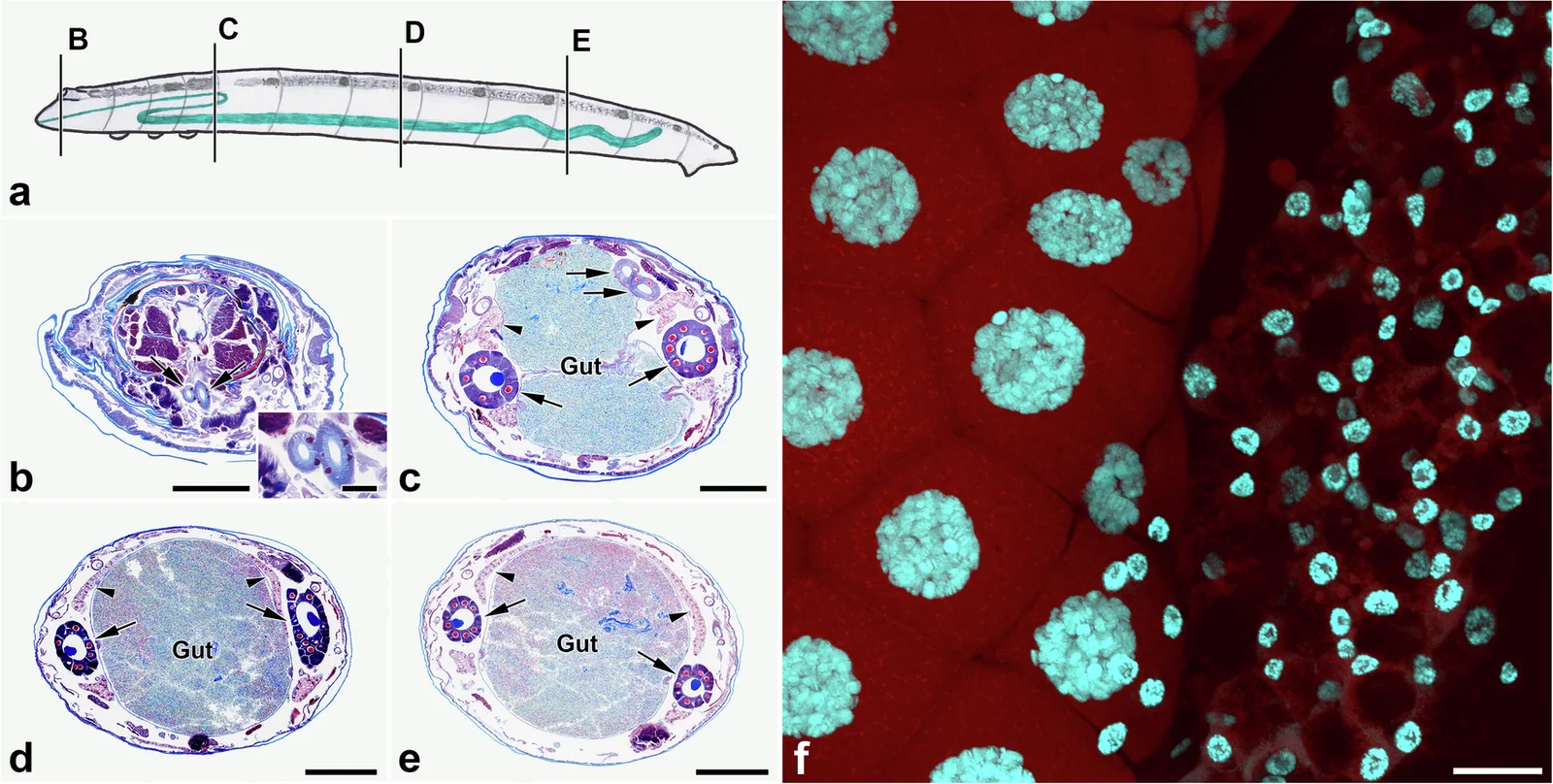
Morphology of the SYGs in *K. testaceus* last-instar larvae. **a** Schematic diagram of a larva showing the general structure of the SYG (green). Black lines labeled B-E correspond to the locations of the cross-sections shown in (**b–e**). **b–e** Paraplast cross-sections stained with Masson trichrome (Sigma). Arrows indicate the SYGs; arrowheads indicate the fat body. **f** DAPI (blue) and phalloidin (red) staining of SYG cells (arrow) reveals enlarged, polyploid nuclei compared to the smaller nuclei of adjacent fat body cells. The diffuse red phalloidin signal results from the confocal z-stack projection of multiple optical sections, causing the actin cortex to appear blurred. To view the actin cortex, see a single optical section in Supplementary Fig. 2. Scale bars: (**b, e**) 200 μm; (**f**) 20 μm.

To investigate the morphology of the salivary glands (SYGs) in *K. testaceus*, we prepared paraplast cross-sections through the body of the last-instar larva. As shown in Fig. 3, the paired tubular SYGs consist of secretory cells extending from the head to the posterior end of the larval body. Anteriorly, the ducts of the left and right glands converge to form a common duct that opens into the labium. The anterior region of the SYGs (ASYG) is narrow and progressively widens into the posterior region (PSYG). At the transition between these two regions, the gland forms a characteristic sigmoid loop. The diameter of the PSYG remains largely constant along its length, tapering only slightly toward the distal end.

DAPI and phalloidin staining revealed that SYG cells contain large, polytene nuclei, a specific form of polyploidy, which were markedly larger than those of adjacent fat body cells. These polyploid cells were distributed uniformly along the entire length of the glands (Fig. 3f and Supplementary Fig. 2).

### Tensile strength of the *K. testaceus* silk

**Fig. 4:**
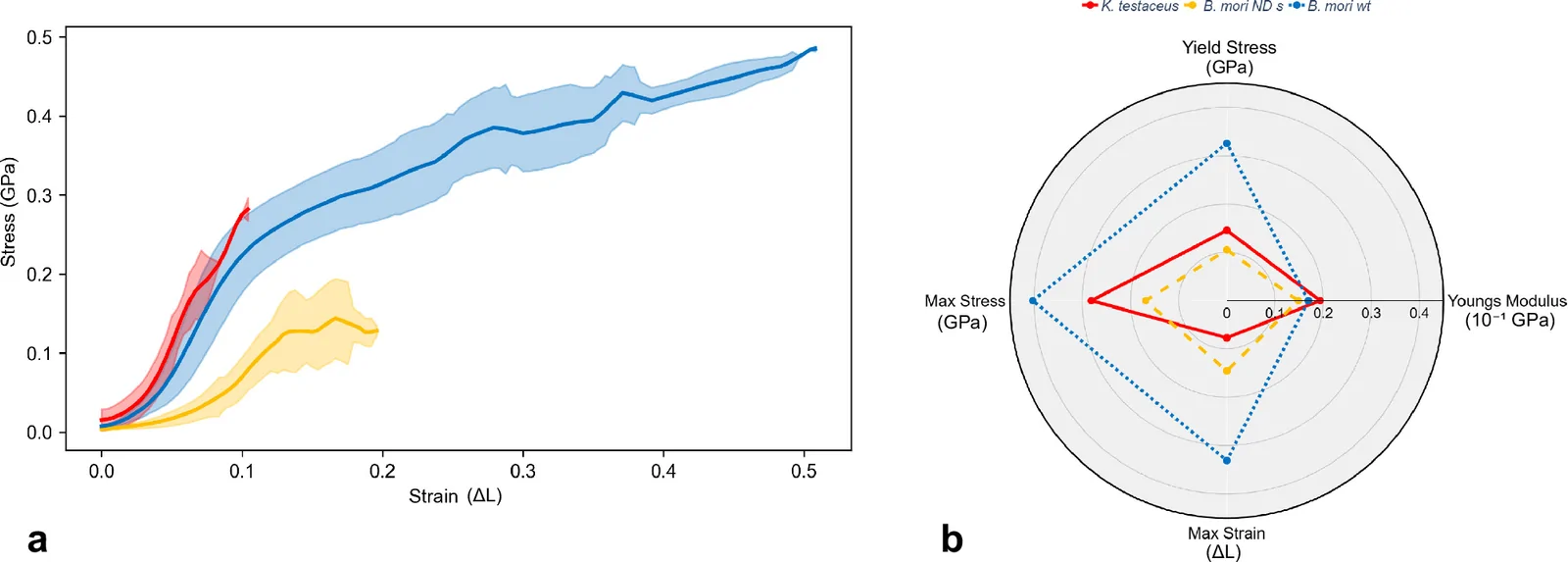
Mechanical properties of silk fibers from *K. testaceus* and *B. mori.* **a** Stress-strain curves showing the average mechanical behavior of *K. testaceus* (red), *B. mori* wt (blue), and *B. mori Nd-s* (yellow). Shaded areas indicate the standard deviation around the mean values (*n* = 10) (Supplementary Fig. 3). **b** Radar plot comparing key mechanical parameters, yield stress, Young’s modulus, maximum stress, and maximum elongation, for the three silk types. Values represent mean ± standard deviation, as summarized in Supplementary Table 1.

To evaluate the structural integrity of *K. testaceus* silk fibers, we compared their mechanical properties with those of *Bombyx mori* wild type (wt) silk and a sericin-rich mutant cocoon silk lacking a fibroin core *(B. mori Nd-s*) (Fig. 4). Due to the challenges in isolating slime-like filaments, only fibers derived from the cocoon, approximately 3 µm in diameter, were tested. These tests were conducted under relatively dry conditions (30% relative humidity (RH)), which typically increases brittleness; however, wet-state testing was not feasible. Mechanical testing assessed yield stress, maximum stress, maximum strain (extensibility), and Young’s modulus. These values are summarized inSupplementary Table 1, with representative stress–strain curves shown in Fig. 4 and Supplementary Fig. 3.

*K. testaceus* silk exhibited a steep, nearly linear stress–strain profile with minimal plastic deformation and an average tensile strength of approximately 230 MPa, suggesting high stiffness but limited extensibility. In comparison, *B. mori* wt silk reached approximately 450 MPa and 50% elongation, demonstrating both strength and ductility. In contrast, *B. mori Nd-s* exhibited the weakest performance across all parameters, indicating a brittle fiber. Compared to *B. mori* wt, the maximum stress values of *K. testaceus* and *B. mori Nd-s* were significantly lower (p < 0.001) (Supplementary Fig. 3).

Young’s modulus was highest for *K. testaceus* and *B. mori* wt, reflecting the steep initial slope of their stress-strain curves. By contrast, *B. mori Nd-s* had a substantially lower modulus, consistent with reduced stiffness.

### Transcriptomic and proteomic analysis of silk in *K. testaceus*

**Fig. 5:**
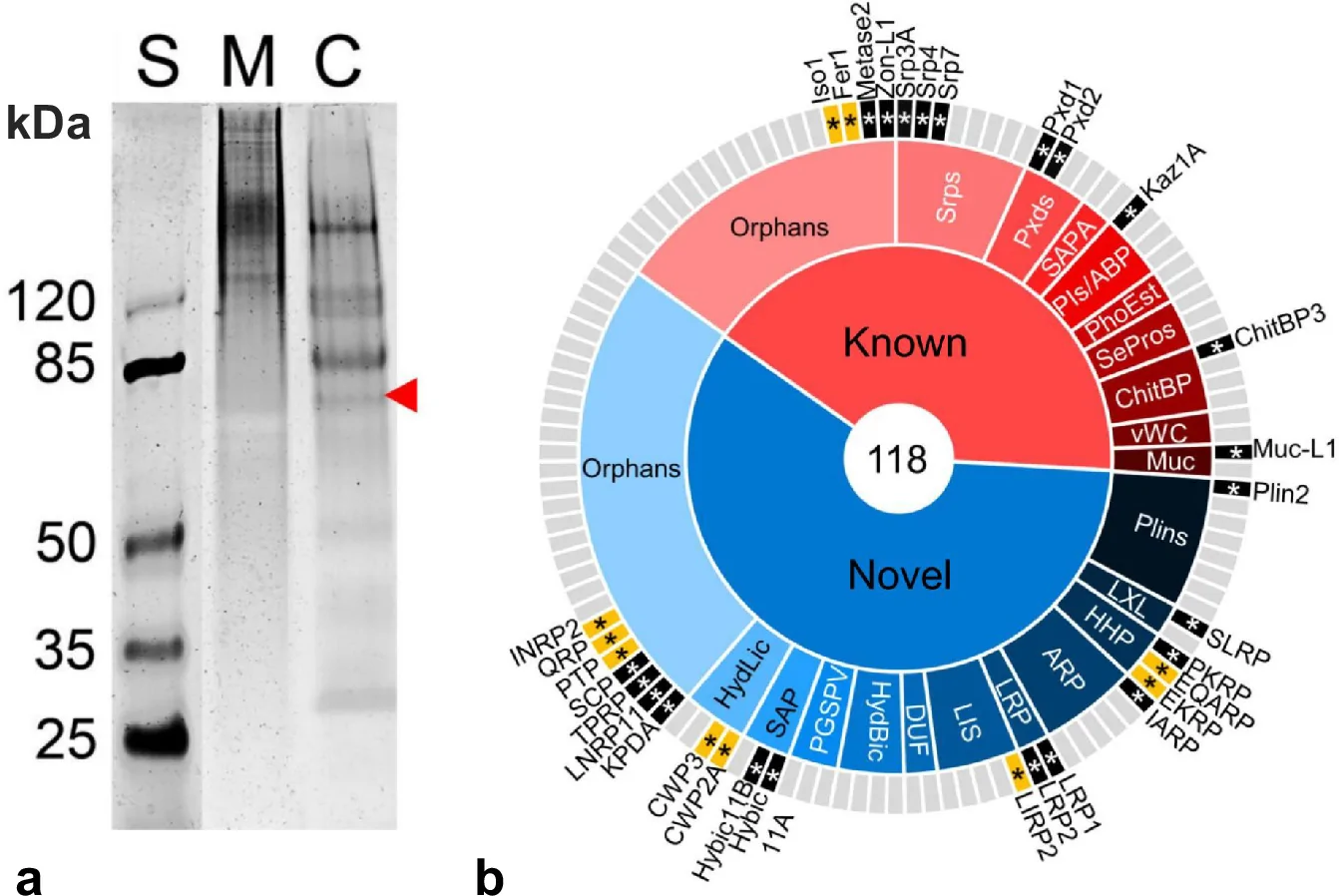
Comparative proteomic profiles of mucus and cocoon in *K. testaceus*. **a** SDS-PAGE comparison of protein profiles of extracts from mucus (M) and cocoon (C). Lane S shows the molecular weight marker. Mucus displays a high molecular weight smear (>120 kDa), while the cocoon shows fewer, distinct bands. The arrow indicates a band at ~78–80 kDa, potentially corresponding to peroxidases discussed in the text. **b** Pie chart illustrating the distribution of 118 secreted proteins into known (with homologs) and novel (no homologs) categories, highlighting relative abundance differences between mucus (yellow) and cocoon (black) proteins. Asterisks (*) indicate statistically significant differences (p < 0.05). Statistical significance was assessed using a two-sample, two-sided Student’s *t*-test. No adjustment for multiple comparisons was applied. Subgroup classifications for known proteins include serpins (Srps), peroxidases (Pxds), saposins (SAPA), protease inhibitors and antibacterial peptides (Pls/ABPs), phosphoesterases (PhoEst), serine proteases (SePros); chitin-binding proteins (ChitinBP), von Willebrand factor type C domain (vWC), and mucin-like proteins **(**Muc). Novel protein groups include prolinins (Plins), leucine-x-leucine motifs (LXL), silk-associated proteins (SAP), highly hydrophilic proteins (HHP), alanine-rich proteins (ARP), leucine-rich proteins (LRP), leucine–isoleucine–serine-rich proteins (LIS), domain of unknown function (DUF), hydrophobic 30 kDa proteins (HydBic), hydrophilic 30 kDa proteins (HydLic) and PGSPV motif proteins (PGSPV). Orphans denote individual proteins that do not belong to any protein family and show no detectable homology to other proteins.

SDS-PAGE analysis revealed distinct protein profiles between mucus and cocoon samples (Fig. 5a). The mucus was dominated by a high molecular weight smear above 120 kDa, consistent with the presence of large, heterogeneous, and heavily glycosylated proteins^14^. In contrast, the cocoon extracts showed several discrete bands at approximately 150, 120, 85, 80, and 30 kDa. Because SDS-PAGE does not allow for specific protein identification, complementary proteomic and transcriptomic analyses were performed to characterize the molecular composition of both silk products.

To identify genes associated with silk synthesis, a cDNA library was constructed from *K. testaceus* larval tissue. De novo transcriptome assemblies were generated using Trinity (49,420 contigs) and SPAdes (25,563 contigs). BUSCO assessment confirmed high assembly completeness, with 93.8% and 94.9% of conserved genes detected in the Trinity and SPAdes assemblies, respectively, supporting the robustness of the transcriptomic dataset.

Proteomic analysis of the cocoon and mucus silk samples identified 118 putative secretory proteins, listed in Table 1 and Supplementary Tables 2A, 2B. Quantitative comparison of protein abundance using Perseus v1.6.15.0^15^ revealed distinct enrichment profiles between the two silk types. In total, 22 proteins were significantly enriched in the cocoon and 10 in the mucus (Fig. 5b). Of these, 12 cocoon-enriched and 8 mucus-enriched proteins corresponded to novel sequences with no identifiable homologs in other organisms. However, no proteins were consistently detected exclusively in either material across biological replicates.

**Table 1:** List of the 28 most abundant candidate silk proteins with their biochemical characteristics

Protein name	Symbol	GenBank ID	Group	Cocoon intensities	Mucus intensities	Secondary structures			No. AA	MW	1^st^ AA(%)	2^nd^ AA(%)	3^rd^ AA(%)	pI	GRAVY
						α(%)	β(%)	dis(%)							
Mucin-like1	Kt_Muc-L1	PX584732	1	33.90*	22.04	6.6	0	93.4	inc.	86	P(15.9)	S(10.0)	T,K(7.7)	9.75	−0.707
Prolinin 2	Kt_Plin2	PX584736	2	33.30*	22.51	2.9	0	97.1	inc.	79	P(18.1)	I(11.1)	S(9.3)	5.64	−0.270
IA rich protein	Kt_IARP	PX584717	2	30.40*	20.90	76.2	4.6	19.2	307	33	I(11.1)	A(10.7)	N(10.4)	5.47	0.176
Serpin 1	Kt_Srp1	PX584754	1	30.10	27.44	20.0	17.8	62.2	inc.	68	P(14.5)	S(12.3)	I(11.5)	4.22	0.019
Prolinin 1	Kt_Plin1	PX584735	2	29.80	29.87	5.8	30.8	63.3	inc.	102	P(12.6)	K(9.0)	L(8.8)	7.26	−0.497
L rich protein 1	Kt_LRP1	PX584729	2	29.40*	21.17	64.1	0	35.9	309	33	L(14.2)	I(10.7)	A(9.7)	4.78	0.383
PK rich protein	Kt_PKRP	PX584734	2	29.20*	20.98	2.3	0	97.7	inc.	204	P(26.7)	K(25.9)	A(13.8)	10.00	−2.082
SL rich protein	Kt_SLRP	PX584752	2	28.70*	23.88	2.5	35.1	62.4	242	26	S(12.4)	L(10.3)	G,Y(8.7)	8.41	−0.072
KPDA tail protein	Kt_KPDA	PX584673	2	28.30*	22.80	7.7	7.0	85.3	414	41	A(27.8)	T(12.6)	P(11.8)	7.62	−0.571
Peroxidase 1	Kt_Pxd1	PX584743	1	28.20*	22.79	n.d.	n.d.	n.d.	692	79	L(9.4)	I(8.7)	N(7.5)	6.09	−0.311
LN rich protein 11 kDa	Kt_LNRP11	PX584676	2	27.90*	20.55	31.7	0	68.3	104	11	L(14.4)	N(12.5)	T(8.7)	6.25	−0.018
Zonadhesin-like 1	Kt_Zon-L1	PX584692	1	27.90*	22.69	20.8	23.2	56.0	327	35	A(12.2)	C(11.3)	N(9.2)	8.32	−0.251
L rich protein 2	Kt_LRP2	PX584730	2	27.30*	22.21	80.3	0	19.7	290	32	L(10.7)	I(9.7)	A,V(9.3)	4.97	0.187
Serpin 3 A	Kt_Srp3A	PX584756	1	27.20*	21.57	27.4	28.8	43.8	413	45	A(10.2)	S,L(9.4)	N,Q(7,3)	4.86	0.063
Serpin 7	Kt_Srp7	PX584761	1	26.90*	21.79	25.9	25.9	48.2	435	47	S(11.0)	L(9.4)	A(8.0)	5.01	0.061
TP rich protein	Kt_TPRP	PX584688	2	26.60*	20.65	31.0	4.0	65.0	200	23	T(11.5)	P(10.0)	I(8.5)	9.75	−0.488
Collagen-like	Kt_Coll-L	PX584703	1	26.30	23.12	0	0.4	99.6	inc.	92	P(26.9)	G(21.6)	V(10.7)	11.4	−0.474
Peroxidase 2	Kt_Pxd2	PX584744	1	26.20*	20.26	n.d.	n.d.	n.d.	304	34	L(8.9)	S(8.2)	N,I(7.6)	7.01	−0.240
Sericin-like	Kt_Ser-L	PX584684	1	26.10	27.40	49.9	0	50.1	515	50	S(23.7)	A(14.8)	Q(12.4)	4.22	−0.143
Metalloaminopeptidase 2	Kt_Metase 2	PX584679	1	26.00*	21.12	45.2	18.5	36.3	940	109	L(9.6)	S(7.7)	E(6.8)	5.77	−0.424
Kazal 1 A	Kt_Kaz1A	PX584722	1	25.80*	20.46	11.2	15.7	73.1	268	29	P(19.0)	S(11.9)	C(7.8)	8.18	−0.462
Chitin-binding protein 3	Kt_ChitBP3	PX584715	1	25.60*	21.15	24.1	12.1	63.8	174	19	A(9.8)	S(9.2)	T,L(8.6)	5.40	0.195
Serpin 4	Kt_Srp4	PX584758	1	25.20*	21.09	28.0	26.6	45.5	429	47	S(11.0)	I(10.7)	L(9.6)	4.85	0.147
EQA rich protein	Kt_EQARP	PX584709	2	23.00	28.49*	52.6	0	47.4	346	40	E(16.8)	Q(14.5)	A(13.3)	8.74	−1.560
CW protein 2 A	Kt_CWP2A	PX584705	2	23.00	28.05*	68.7	2.9	28.4	275	32	L(11.6)	S(8.4)	I,N(7.6)	8.99	−0.155
EK rich protein	Kt_EKRP	PX584708	2	22.20	28.50*	40.7	0	59.3	560	65	E(20.7)	K(18.4)	A(10.9)	6.82	−2.016
Isomerase 1	Kt_Iso1	PX584776	1	20.30	24.78*	11.4	26.7	61.9	202	22	G(13.4)	V(9.4)	A(8.9)	6.85	−0.066
CW protein 3	Kt_CWP3	PX584707	2	19.60	25.30*	78.7	0	21.3	267	30	L(11.6)	N(10.5)	S(9.7)	6.33	−0.206

*Group1* - proteins with known homologs, *Group2 *- novel proteins, *α *- alpha helices, *β *- beta strands, *dis *- disordered regions, *n.d*. - not determined. *No, AA* – number of amino acids, *MW*– molecular weight (kDa), *AA* – amino acid composition, *pI* – isoelectric point, *GRAVY* – grand average of hydropathy, *inc.* – properties calculated from partial sequences. Protein iBAQ intensities were log₂-transformed for statistical analyses (two-sided t-test). Asterisks indicate proteins significantly enriched in the respective silk material (p < 0.05). Exact p-values are provided in Supplementary Tables 2A and 2B.

### Putative silk proteins and their annotation

The identified secretory proteins were classified into two main categories: (1) proteins with recognizable homologs based on sequence similarity to proteins in other species, and (2) novel proteins that lack identifiable homologs or conserved domains. The first category included 49 sequences that could be assigned to functional groups such as mucin-like, collagen-like, sericin-like, and zonadhesin-like proteins, as well as several smaller protein families. These included serpins, serine proteases, Kazal-type protease inhibitors, peroxidases, and proteins containing chitin-binding domains.

Another major cocoon component exhibited a repetitive Gly–X–X motif and was strongly enriched in proline (26.9%). This protein was therefore designated collagen-like (Coll-L) (Supplementary Fig. 4). Additional abundant proteins included sericin-like proteins, serpins, peroxidases, chitin-binding proteins, saposins, and various enzymes with annotated domains, such as sphingomyelin phosphodiesterase, metallophosphatase, glucosylceramidase, metalloaminopeptidase, glycohydrolase, isomerase, and histidine phosphatase (Supplementary Table 3). The sericin-like proteins were particularly rich in serine (23.7%) and alaine (14.8%) residues (Table 1).

The second category comprised 69 novel, species-specific secretory proteins, forming a large and diverse group. Most of these were grouped into small protein families as shown in Table 1 and Supplementary Table 2B. The most abundant members of this category were proline-rich, highly hydrophilic proteins.

These included a highly basic PK-rich protein (proline-lysine) containing 26% proline, and a small group of eight proteins designated as prolinins (Plins). These Plins represent proline-rich secreted proteins containing 10–18% proline, lacking recognizable conserved domains or detectable homologs outside this species, and predicted to be largely disordered (Supplementary Table 2B). Two additional abundant proteins were hydrophobic and named based on dominant amino acid composition: an IA-rich protein and an L-rich protein 1. A complete list of these abundant Group 2 proteins, along with their proposed classifications, is provided in Supplementary Table 2B.

### SYG specificity of candidate silk proteins

**Fig. 6:**
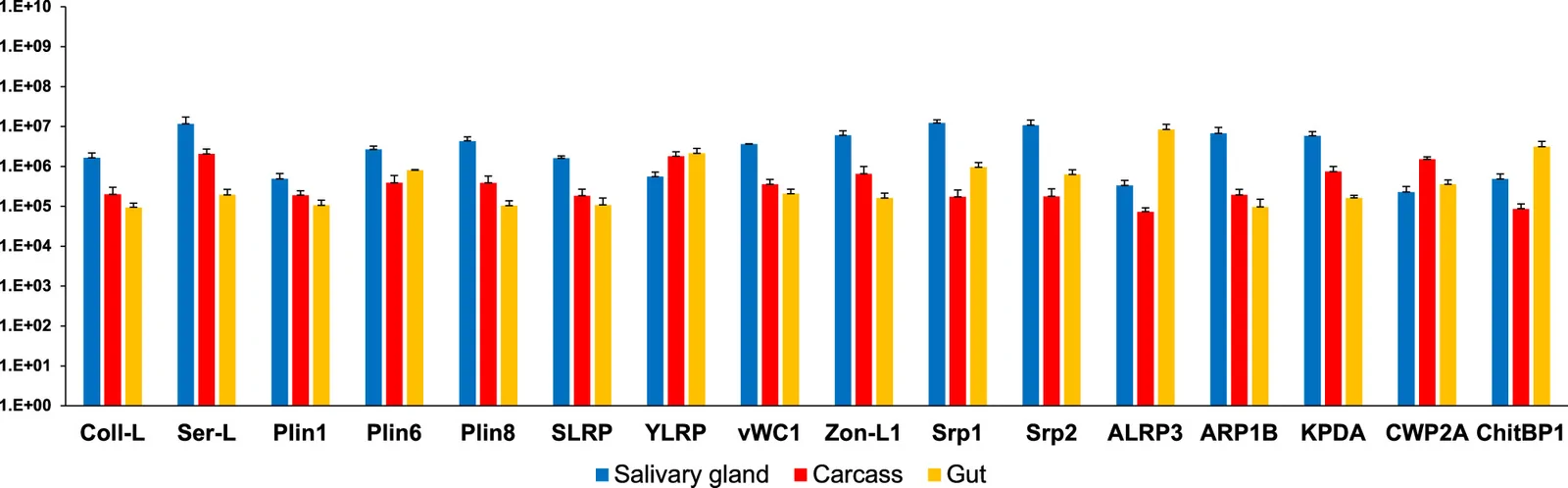
Quantitative PCR analysis of 16 selected genes in *K. testaceus*. Twelve genes (*Coll-L, Ser-L, Plin1, Plin6, Plin8, SLRP, vWC1, Zon-L1, Srp1, Srp2, ARP1B*, and *KPDA*) show markedly higher expression in the SYGs compared to the gut and carcass. The remaining four genes (*ALRP3, ChitBP1, YLRP*, and *CWP2A*) did not show predominant expression in the SYGs. Gene symbols correspond to the protein names listed in Tab. 1 and Supplementary Tables 2A and 2B. Expression values (2⁻ΔCt) were scaled by 10⁵ for visualization on a logarithmic scale. Data are presented as mean ± SEM (*n* = 3 biological replicates, each measured in technical triplicates; each replicate represents an independent larva). Overall differences in gene expression among tissues were evaluated using the Kruskal–Wallis test (*p* < 0.05)^50^. Pairwise comparisons were performed using Wilcoxon tests^51^.

To confirm the tissue specificity of transcription for selected candidate silk genes in the SYGs of *K. testaceus*, quantitative real-time PCR (qRT-PCR) was conducted using RNA extracted from the SYGs, intestines, and carcass tissues. The results are presented in Fig. 6.

Among the 16 genes analyzed, 12 exhibited significantly higher expression levels in the SYGs (*p* < 0.05). N These included *Coll-L, Ser-L, Plin1, Plin6, Plin8, SLRP, vWC1, Zon-L1, Srp1, Srp2, ARP1B*, and *KPDA*, all of which showed markedly elevated expression in the SYGs compared to the gut and carcass.  None of the analyzed transcripts were exclusively expressed in a single tissue. In contrast, *ALRP3* and *ChBP1* were primarily expressed in the intestine, while *YLRP* and *CWP2A* showed elevated expression in both the carcass and intestine, suggesting broader physiological functions.

### Differences between mucus web and cocoon

SEM revealed clear structural differences between the mucus web and cocoon silk of *K. testaceus*. The cocoon exhibited a well-defined framework of fiber-like structures that were optically denser than the surrounding matrix, in contrast to the more diffuse appearance of the mucus web.

Proteomic analysis reinforced these observations by revealing pronounced differences in protein composition between the two silk types. Several proteins, including Plin 2, Muc-like 1, PK-rich protein, IA-rich protein, L-rich proteins 1 and 2, and LN-rich protein, were highly enriched in cocoon silk, with enrichment levels in some cases reaching up to three orders of magnitude higher than in the mucus web.

### Protein secondary structures - analysis and prediction

**Fig. 7:**
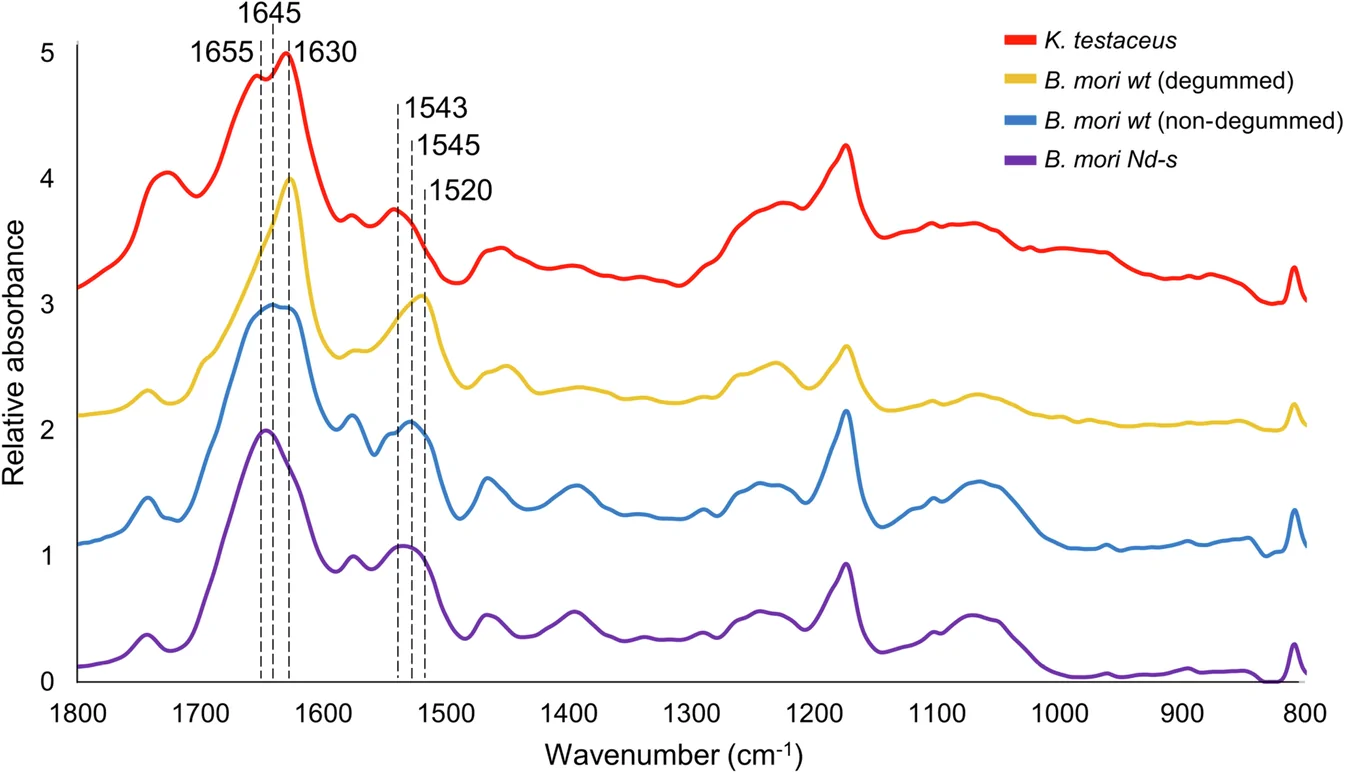
FTIR spectra of K. testaceus cocoons compared with those of *B. mori*. Spectra are shown for silk fibers from *K. testaceus* (red), degummed *B. mori*wt (yellow), non-degummed *B. mori* wt (blue), and *B. mori Nd-s* (violet). The spectra have been vertically offset for clarity. The amide I region (1600–1700 cm⁻¹) shows main absorption peaks at 1655, 1645, and 1630 cm⁻¹, while the amide II region (1500–1600 cm⁻¹) displays peaks at 1545, ~1543, and 1520 cm⁻¹.

To investigate the secondary structure composition of *K. testaceus* silk proteins, we recorded FTIR spectra of four silk samples: *K. testaceus*, *B. mori* wt (with and without degumming), and *B. mori Nd-s*, a fibroin-deficient mutant. All spectra showed distinct variations in the amide I (1600–1700 cm⁻¹) and II region (1500-1600 cm⁻¹), which are highly sensitive to protein secondary structures (Fig. 7). *K. testaceus* silk exhibited a dominant amide I peak at ~1630 cm⁻¹ with a secondary maximum near 1655 cm⁻¹. The amide II region showed strong absorption around 1543 cm⁻¹.

To identify candidate proteins responsible for these ordered structures, we predicted the secondary structures of the most abundant *K. testaceus* silk proteins using AlphaFold, complemented by analysis with the 2Struc secondary structure server^16,17^. The predicted proportions of α-helices, β-strands, and disordered regions are summarized in Table 1. These results indicate that many *K. testaceus* silk proteins contain a substantial proportion of β-strands, along with notable levels of α-helices and intrinsically disordered regions. As shown in Supplementary Fig. 5, the PK-rich protein, the central region of mucin, and prolinin 8 are likely to contain PPII helices.

## Discussion

In this study, we characterized the SYGs of *K. testaceus* and their proteinaceous secretions using histology, electron microscopy, mechanical testing, transcriptomics, proteomics, and FTIR spectroscopy. The larvae produce two distinct secretions: a mucus web during the larval stages and a solid cocoon at the end of the final instar. These materials differ in morphology and protein composition, which is consistent with their distinct functional roles.

Most silk proteins identified in *K. testaceus* are novel, showing no significant similarity to any previously characterized proteins. Notably, the silk secretions of *K. testaceus* contain no fibroin subunits, which is consistent with prior evidence that fibroins are specific to Lepidoptera and Trichoptera silk systems^2^.

In Diptera, larval SYG secretions are highly species-specific, reflecting adaptations to diverse ecological niches^18–20^. For example, larvae of *B. coprophila* secrete large amounts of chitinases, enzymes likely involved in degrading fungal and plant cell walls as part of their detritivorous feeding strategy^21^. In contrast, the predatory keroplatid glowworm *A. luminosa* produces silk-based fishing lines and nests primarily composed of hydrophilic proteins with a highly disordered structure. Proteomic analyses of this silk have identified long, hydrophilic proteins rich in lysine and proline, along with smaller protease inhibitors^8,9^. Notably, *A. luminosa* silk fibers are strong, flexible, and elastic when hydrated, but become brittle and inelastic upon drying^22^. Similarly, *K. testaceus* silk is composed predominantly of hydrophilic proteins, and its mechanical properties are likely influenced by hydration.

Our results reveal marked differences between the mucus web and the cocoon of *K. testaceus*, most likely reflecting functional specialization. The cocoon silk contained a greater proportion of proline-rich proteins, which are likely involved in hydration maintenance and cohesive molecular interactions among silk components. Additionally, the higher abundance of hydrophobic proteins (e.g., IA-rich-, L-rich, and LN-rich proteins), along with elevated levels of peroxidases, could contribute to the structural stabilization and water-resistant properties of the cocoon. These compositional differences likely account for the distinct mechanical and functional characteristics of the two silk types. It should be noted that further mechanical measurements under native hydrated conditions are needed, as the current method may have affected the results for fibers that are normally highly hydrated.

The mucus web appears adapted for short-term use, emphasizing flexibility and adhesion - properties that facilitate substrate attachment and the capture of fungal spores (Supplementary Movie). In contrast, the cocoon material appears to be adapted for mechanical strength and moisture retention, ensuring protection during pupation. Cocoon formation is accompanied by the upregulation of numerous abundant proteins accompanies cocoon formation in addition to those employed in mucus production, reminiscent of the developmental switch in *Drosophila melanogaster* larvae from food-associated salivary proteins to glue proteins during pupariation^23^.

Proteomic analysis of *K. testaceus* cocoon silk further revealed the presence of several peroxidases, including three isoforms that were largely absent in the mucus web. A prominent 78–80 kDa band (Fig. 5a) likely corresponds to these enzymes. In caddisfly silk, such enzymes stabilize the cocoon via dityrosine cross-linking, thereby enhancing mechanical strength and water resistance^24^. Additionally, multiple protease inhibitors were detected, suggesting that the silk matrix may be protected from enzymatic degradation during the pupal stage. Similar findings have been reported in lepidopteran and trichopteran silk^24,25^. Chitin-binding proteins, which were found in both the cocoon and mucus web, may facilitate adhesion to fungal substrates or other environmental surfaces^26^.

One of the most striking features of *K. testaceus* silk is its high content of proline-rich proteins (PRPs) in both the cocoon and the mucus web. The collagen-like protein (Coll-L exhibits a regular arrangement of GVP or GQP motifs, characteristic of collagen-like molecules. The repetitive domain of this collagen-like protein shares 40% sequence identity with the (GXY, frequently Y = Pro) repeats of human collagen (Supplementary Fig. 4), known to form a triple helix structure. The notably high proportion of proline residues in *K. testaceus* collagen-like protein is comparable to that observed in the silks of hymenopteran sawflies, such as the gooseberry sawfly (*Nematus ribesii*) and the willow sawfly (*Nematus oligospilus*). In these insects, such collagen-like motifs contribute to silk strength, flexibility, and stability^27–29^.

In addition to collagen-like proteins, *K. testaceus* silk contains several other abundant PRPs with less regular repeat patterns. These proteins appear to be much more prevalent in the cocoon silk than in the mucus web. Notably, proteins with PXY repeats - such as prolinin 2, mucin-like 1, and other prolinins - are predicted to contain intrinsically disordered regions (Tab. 1 and Supplementary Tables 2A and 2B) with the potential to form PPII helices.

PRPs with similar structural motifs and intrinsically disordered regions have also been identified in moth and spider silks. For example, seroins from various moths contain approximately 20% irregularly arranged proline residues and are suggested to function as antimicrobial proteins^30^. In spiders, major ampullate spidroin 2 of *Araneus ventricosus*, which contains 13% proline residues, features repeating GPG motifs^31^, whereas the flagelliform silk of *Nephilengys cruentata*, with 17.4% proline residues, exhibits GPGGX motifs^32^. These repetitive motifs are known to contribute substantially to the remarkable elasticity of spider silk fibers.

Interestingly, comparable proteins bearing similar proline-rich domains are also found in mammals. In humans, PRPs are major constituents of saliva, where they perform diverse functions including microbial regulation, surface adhesion, lubrication, and interactions with dietary polyphenols such as tannins^33^. Another example is amelogenin, a proline-rich protein that constitutes the predominant extracellular matrix component during mammalian tooth enamel formation. Amelogenin self-assembles into nanospheres within the extracellular matrix, thereby guiding the organization and structural development of enamel^34^.

This functional resemblance may indicate a case of convergent evolution, in which PRPs have independently evolved to enhance structural integrity and mediate environmental interactions - such as moisture retention and microbial control - in both insects and vertebrates.

Our study found that FTIR spectra of *K. testaceus* cocoons showed distinct variations in the amide I region (1600–1700 cm⁻¹) (Fig. 7). Three characteristic absorption bands were observed near 1655, 1645, and 1630 cm⁻¹, corresponding to C = O stretching vibrations and indicative of α-helix, random-coil/disordered conformations, and β-sheet–rich crystalline domains, respectively^35^.

The spectrum of *K. testaceus* silk exhibited a strong, sharp band centered at approximately 1630 cm⁻¹, characteristic of high β-sheet content and a well-ordered, crystalline structure^36^. A secondary maximum near 1655 cm⁻¹ suggests the presence of α-helical and random coil elements. Several proteins with a high predicted propensity for β-sheet formation, including prolinins 1, 3, 4, 5, and 7, as well as serpin 1, SL-rich, and SLRP-rich proteins, were detected in *K. testaceus* silk. However, based on proteomic sequence analysis revealing a high content of proline-rich components, the presence of triple helical structures or PPII helices, such as those found in collagen, is also plausible. In contrast*, B. mori* wt silk displayed a broader amide I peak with local maxima at 1630 and 1645 cm⁻¹, consistent with a mixed structural composition comprising β-sheet–rich fibroin and disordered or helical sericin regions^36^. This interpretation was supported by spectra from the *B. mori Nd-s* mutant (fibroin-deficient), which showed a main band shifted toward 1645 cm⁻¹ and reduced β-sheet signal intensity, indicating the predominance of sericin-rich, disordered conformations. Conversely, degummed *B. mori* silk, representing purified fibroin, exhibited a sharper amide I band centered approximately at 1630 cm⁻¹, consistent with a dominant β-sheet structure^36^. This interpretation of the FTIR spectra (Fig. 7) was confirmed by Fourier self-deconvolution (FSD) of the amide I region, which is widely applied to quantify secondary structural elements in silk materials^36^ (supporting information Supplementary Figs. 6, and 7). Consistent with the sharp 1630 cm⁻¹ band observed in the native spectrum, degummed *B. mori* silk exhibited the highest β-sheet content (37.2%), reflecting the predominance of highly ordered fibroin crystalline domains. In contrast, the *B. mori Nd-s* mutant showed a markedly reduced β-sheet fraction (26.4%) together with elevated random coil (31.2%) and α-helical content (13.0%), confirming the dominance of sericin-rich, less ordered conformations in the absence of fibroin. Non-degummed *B. mori* wt displayed an intermediate structural profile (30.1% β-sheet; 28.3% random coil; 24.0% turn), consistent with its composite architecture of crystalline fibroin embedded within a more amorphous sericin matrix. Notably, *K. testaceus* silk exhibited a comparatively balanced distribution of secondary structures, with β-sheet (28.2%) and random coil (29.8%) contributions of similar magnitude, accompanied by a substantial turn content (21.1%) and moderate α-helical fraction (12.4%). Although its β-sheet content did not exceed that of degummed *B. mori*, the narrower spectral features and distinct peak distribution suggest a structurally organized yet less fibroin-dominated architecture. The relatively high proportion of turn and helical components in *K. testaceus* may reflect the presence of proline-rich and repetitive sequence motifs identified in the proteomic analysis, which are known to favor β-turns, polyproline II (PPII)-type conformations, or triple-helical arrangements rather than extended β-sheet crystallites.

A similar trend was observed in the amide II region (1500–1600 cm⁻¹), which corresponds to the asymmetric combination of C–N stretching and N–H bending. β-sheet–rich structures typically absorb at lower wavenumbers (1530–1540 cm⁻¹), while disordered and helical conformations absorb at higher frequencies (1545–1550 cm⁻¹)^35^. The *K. testaceus* silk spectrum showed strong amide II absorption in the higher portion of the region (1543 cm⁻¹), supporting the presence of substantial helical content. The degummed *B. mori* sample exhibited lower amide II frequencies (1520 cm⁻¹), consistent with β-sheet–rich material, whereas both the non-degummed *B. mori* wt and *B. mori Nd-s* samples displayed broader bands centered approximately at 1545 cm⁻¹, indicating a predominance of amorphous or helical secondary structures.

Previous studies of glowworm (*A. luminosa*) silk have also reported substantial β-sheet content in the fibers, with X-ray scattering confirming the presence of cross-β-sheet crystallites in the dry state^22^. Glowworms and *K. testaceus* share a close evolutionary relationship, and similar stress–strain responses often reflect comparable molecular architectures^37^. The similar mechanical behavior of glowworm and *K. testaceus* silk under dry conditions may therefore be consistent with the presence of cross-β-sheet structures in *K. testaceus* silk.

The *K. testaceus* silk spectra supported the presence of substantial helical content. However, it remains speculative to distinguish between α-helix, PPII, and triple-helical structures solely based on this spectral feature. FTIR and in silico structural prediction need to be supplemented with other high-resolution methods. Future work should investigate the structural organization and post-translational modifications of these proline-rich proteins, assess the mechanical behavior of mucus-derived filaments under native hydrated conditions, and functionally characterize low-abundance and unannotated proteins to clarify their contributions to silk assembly and material performance.

In summary, these results suggest that *K. testaceus* has evolved a fibroin-independent silk system in which hydrophilic, collagen-like, β-sheet-rich, and accessory proteins collectively contribute to the material’s strength, elasticity, adhesion, and environmental resilience. This system produces two functionally distinct silk types, each supported by slightly different protein repertoires. The mucilaginous silk, characterized by collagen-like structures, facilitates larval mobility, substrate attachment, and feeding via spore collection, while the cocoon silk, composed of mixed β-sheet, α-helix, or triple-helical collagen elements, provides mechanical support and protection during pupation and the entire pupal stage. The exceptional stretchability and adhesive properties of mucilaginous silk may provide insight into natural hydrogel-like materials. The unique combination of hydrophilic, proline-rich, collagen-like, and β-sheet–forming proteins in *K. testaceus* demonstrates an alternative natural strategy for producing hydrated yet mechanically robust materials, providing a foundation for future structural and functional investigations of this silk system.

## Methods

### Biological material

Larvae of *K. testaceus* were collected from the underside of partially decayed conifer logs and from the pore surfaces of mature bracket fungi on conifer stumps near Černé Údolí, Czech Republic (48°41′40.92″ N, 14°41′46.42″ E). In captivity, larvae produced mucus and spun cocoons, which were collected and analyzed. Species identity was confirmed by DNA barcoding of the mitochondrial *cytochrome c oxidase subunit I* (*COI*) gene (GenBank accession no. PV206836.1), using the BOLD Identification System. In addition, the COI sequence was compared with the reference sequence of *Keroplatus tipuloides* (MT446888.1), a morphologically indistinguishable species at the larval stage (Supplementary Fig.  8).

Whole larvae were photographed using a Nikon D5600 digital camera equipped with an AF-S Micro Nikkor lens (Nikon Corporation). High-resolution Z-stack images of larvae and cocoons were acquired using an SZX16 stereomicroscope (Olympus, Tokyo, Japan) equipped with a PROMICAM 3-5CP camera (Promicra, Prague, Czech Republic). Image stacks were merged using Quick Photo Camera 3.1 software. Silk from *B. mori* wt and the mutant strain *B. mori Nd-s*, which lacks a fibroin core, was used as reference materials for comparative FTIR and tensile strength analyses. These silk samples were generously provided by Dr. Hidetoshi Teramoto (NARO, Tsukuba, Japan).

All field collections of *K. testaceus* larvae and cocoons were performed in accordance with regional conservation regulations and did not involve any protected species. Because work was conducted exclusively on wild invertebrates, no institutional ethical approval was required under Czech or EU legislation. All procedures complied with standard ethical guidelines for the use of invertebrates in research.

### Scanning electron microscopy

Mucus web specimens were fixed in 1% osmium tetroxide for two weeks at −20 °C. Cocoons of *K. testaceus* were air-dried. Samples were mounted onto aluminum holders, sputter-coated with a thin layer of gold, and examined for ultrastructural features using a field-emission scanning electron microscope (JEOL JSM-7401F; JEOL, Akishima, Japan).

### Histology

The cuticle of water-anesthetized *K. testaceus* larvae was punctured with a fine needle under Bouin-Hollande fixative solution supplemented with mercuric chloride^38^, and tissues were fixed overnight at 4 °C. Standard histological procedures included dehydration, paraffin embedding, sectioning (7 μm), deparaffinization, and rehydration. Sections were treated with Lugol’s iodine followed by a 7.5% sodium thiosulfate solution to remove residual heavy metals, rinsed in distilled water, and stained using the HT15 Trichrome (Masson) staining kit (Sigma-Aldrich, Inc., St. Louis, MO, USA) according to the manufacturer’s protocol. The stained sections were dehydrated and mounted in DPX mounting medium (Fluka, Buchs, Switzerland). High-resolution images were acquired using a BX63 microscope with a DP74 CMOS camera, and composite images were stitched using CellSens software (Olympus).

### Visualization of polyploid nuclei in SYG

Larval SYGs with attached fat bodies were dissected in sterile phosphate buffer (PB) and fixed in 4% formaldehyde in PB for 20 min at 20-25 °C. Following three 10-minute washes in PB, filamentous actin (F-actin) was stained using Phalloidin-Atto 565 (Sigma-Aldrich, Merck KGaA, Darmstadt, Germany). After an additional three 10-minute PB washes, tissues were mounted in Fluoroshield with DAPI (Sigma-Aldrich, Merck KGaA, Darmstadt, Germany). Stained tissues were visualized using a FluoView FV3000 confocal laser scanning microscope (Olympus Corporation, Tokyo, Japan).

### Sodium dodecyl sulfate–polyacrylamide gel electrophoresis (SDS-PAGE) and silver staining

Equal amounts of mucus and cocoon samples (approximately the size of a small pinhead) were dissolved in 25 μl of sample loading buffer (10 mM Tris-HCl pH 6.8, 1% SDS, and 1% 2-mercaptoethanol) and heated at 96 °C for 10 min. The solubilized silk proteins were separated by 10% Tris–Tricine SDS–PAGE, as described by Haider^39^. Proteins were visualized via silver staining using the protocol of Kirkeby^40^ and the stained gels were imaged using a GS-800 Calibrated Densitometer (Bio-Rad).

### Extraction of RNA and construction of transcriptomes

Total RNA was extracted from last-instar larvae using TRIzol reagent (Life Technologies, Carlsbad, CA, USA) according to the manufacturer’s instructions. RNA was isolated and prepared for sequencing on an Illumina platform using a KAPA Stranded mRNA-Seq Kit and magnetic oligo(dT) beads to capture poly(A)-tailed mRNA. cDNA libraries were prepared following the protocol of Rindos^41^ and sequenced on a MiSeq system (Illumina, San Diego, CA, USA) to generate 2 × 150 bp paired-end reads. Transcriptomes were assembled using Trinity (Galaxy v. 2.15.1+galaxy1) and RNA SPAdes (Galaxy v. 4.2.0+galaxy0) on the Galaxy platform. Transcriptome completeness and quality were assessed using BUSCO (Galaxy v. 5.5.0 +galaxy0, insecta _odb10 database).

### LC-MS analysis and data processing

Four cocoons and four mucus samples were dissolved in 8 M urea, sonicated using a Bandelin Sonoplus Mini 20 (MS 1.5), and the protein concentration was measured using the BCA protein assay kit (Sigma-Aldrich). Ten micrograms of protein was diluted 10 times by 2% sodium deoxycholate (SDC) and 100 mM triethylammonium bicarbonate (TEAB). The samples were afterward mixed with 40 mM chloroacetamide (CAA) and 10 mM tris(2-carboxyethyl)phosphine (TCEP) and kept for 30 min at 40 °C. After the reduction and alkylation step, samples were processed with SP3 beads following the protocol of Hughes^42^. Proteins were digested with trypsin in 100 mM TEAB. The resulting peptides were acidified with trifluoroacetic acid (TFA), 1% final concentration, and desalted using in-house C18 StageTips^43^. Peptide mixtures were analyzed by nano–LC–MS. Separation was performed on an Aurora Ultimate TS column (25 cm × 75 μm, 1.7 μm; Ion Optics) using a nano-reversed phase system. Mobile phases consisted of solvent A (water with 0.1% formic acid) and solvent B (acetonitrile with 0.1% formic acid). Samples were initially trapped on a C18 PepMap100 column (5 μm, 300 μm × 5 mm; Thermo Scientific) for 4 min at 18 μl/min in 2% acetonitrile with 0.1% TFA, then eluted with a 4–35% gradient of solvent B over 60 min. Peptides were analyzed using a Thermo Orbitrap Ascend mass spectrometer operated in DIA mode. Survey scans (350–1400 m/z) were acquired at 60,000 resolution (at 200 m/z) with an AGC target of 4 × 10⁵ ions. DIA scans were acquired at 30,000 resolution with an AGC target of 1000% and automatic injection time. The 400–1000 m/z range was covered using 30 × 20 Da windows, and HCD collision energy was set to 28%.

Raw files were processed using MaxQuant v1.6.17.0^44^. Database searching was performed against a custom protein database generated from RNA sequencing data. Trypsin/P was specified as the proteolytic enzyme, allowing up to two missed cleavages. Carbamidomethylation of cysteine was set as a fixed modification, while oxidation of methionine and protein N-terminal acetylation were included as variable modifications. The minimum peptide length was set to seven amino acids. Peptide and protein identifications were filtered at a false discovery rate (FDR) of 1% using a target-decoy approach. Identified proteins were manually annotated using tblastn (BioEdit).

### Expression validation by qRT-PCR

To verify tissue-specific mRNA expression in the SYGs, whole-body carcasses, SYGs, and intestines were dissected for mRNA isolation and subsequent qRT-PCR. Reactions (20 μl) contained 5 μl diluted cDNA, 250 nM of each primer, and HOT FIREPol EvaGreen qPCR Mix Plus (Solis BioDyne, Tartu, Estonia). Amplification was performed under the following conditions: initial denaturation and polymerase activation at 95 °C for 12 min, followed by 40 cycles of 95 °C for 15 s, primer-specific annealing for 20 s, and 72 °C for 20 s. Melting curve analysis was conducted to assess amplification specificity by ramping from 75 °C to 95 °C in 1 °C increments every 5 s to confirm a single specific amplicon. Primers (Supplementary Data 1) were designed using Primer3Plus software^45^. Data were analyzed and quantified using Rotor Gene Q software (v.2.3.5). Each biological sample was measured in technical triplicate, and statistical analyses were performed on three independent biological replicates.

### Prediction of silk protein structures

The structure and potential function of the most abundant protein sequences identified from cocoon and mucus samples were predicted using AlphaFold2 (Galaxy v. 2.3.2+galaxy4)^16,17,46^. Secondary structure elements were further analyzed using the 2Struc server with the DSSPcont algorithm to assign and refine α-helices, β-sheets, and disordered regions^46^. Polyproline II helices were predicted using PPIIpred^47^ and data visualization was performed in R 4.5.2 using stringr 1.5.1, tidyr 1.3.1, dplyr 1.1.4, and ggplot2 3.5.2. Predicted 3D structures are provided in Supplementary Data 2.

### FTIR spectroscopy

To determine the secondary structure in cocoon proteins, we used FTIR spectroscopy with attenuated total reflectance (ATR). Measurements were performed using a Bruker Tensor 27 spectrometer (Bruker, Germany) equipped with a germanium crystal. Spectra were acquired in ATR mode at a resolution of 4 cm^−1^ across the range of 4000–800 cm^−1^, averaging 100 scans per spectrum. Atmospheric compensation was applied to correct for fluctuations in water vapor and carbon dioxide. Due to the natural surface roughness of the cocoons (tens of micrometers), which can reduce contact with the ATR crystal, an anvil was used to ensure consistent and reliable sample contact. Spectra were recorded from four different locations on the outer surface of each cocoon. This procedure was conducted on cocoons of *K. testaceus*, *B. mori*-wt, and *B. mori Nd-s* mutants (which contain sericin but lack fibroin). After degumming in 0.4% sodium bicarbonate solution for 2 h at 96 °C, both *K. testaceus* and *B. mori Nd-s* cocoons dissolved completely and could not be analyzed post-treatment. In contrast, degummed *B. mori* wt cocoons remained intact and were successfully analyzed. All spectra were baseline-corrected and normalized using OPUS software (version 8.0).

FSD of the amide I region (1590–1705 cm⁻¹) was performed using OPUS software (Bruker Optics). Prior to analysis, the spectra were baseline-corrected and smoothed. Spectral narrowing was achieved by applying Lorentzian line shapes with a half-bandwidth of 23 cm⁻¹ and a noise-reduction factor of 0.3 within the 1590–1705 cm⁻¹ range. The resulting deconvoluted amide I envelopes were then subjected to peak fitting using Gaussian functions. The initial peak positions were assigned according to the ranges established in the literature: side chains (1595–1615 cm⁻¹), β-sheets (1616–1637 cm⁻¹ and 1697–1703 cm⁻¹), random coils (1638–1655 cm⁻¹), α-helices (1656–1662 cm⁻¹) and turns (1663–1696 cm⁻¹). Curve fitting was first carried out using a local least-squares approach with fixed bandwidths. This was then followed by iterative refinement using the Levenberg–Marquardt algorithm with variable bandwidths to optimize peak parameters. FSD analysis was conducted on three to four independent samples of each silk type.

### Tensile strength measurement

To assess whether the mechanical properties of *K. testaceus* silk fibers correspond to their biological function, we conducted mechanical tensile testing on silk fibers with a diameter of approximately 3 µm under controlled conditions (30% relative humidity). The testing setup included a tensometric transducer with 200 g capacity, interfaced via an HX711 processing module and a 24-bit AD converter. This was connected to an NAE016 stepper motor using an L298N H-bridge (Geekcreit Dual H-Bridge Driver Board)^48^. Given the fragility of individual fibers, three fibers were arranged in parallel and fixed onto a 10 mm paper disk for mechanical stabilization. The mounted fibers were connected to a lab-scale balance weight and a string looped over a fine pulley attached to the rotating motor. The measuring length was 3 mm, and the elongation rate was kept constant at 0.025 mm per step. Force changes were recorded with a resolution of 0.01 g. System operation and data acquisition were managed via an Arduino Nano microcontroller connected to a PC through a USB interface. The data were captured using proprietary ForceSketch software. Measurements were taken until fiber breakage. Force data were normalized to the cross-sectional area of the fiber bundle (mm²), and stress values were calculated and expressed in GPa. Fiber diameters were measured prior to testing using an Olympus BX63 wide-field optical microscope. For each sample, diameters were measured at three distinct positions per image using FIJI (ImageJ) software^49^. Force-displacement data, together with cross-sectional measurements, were used to derive engineered stress-strain curves, from which yield point, maximum stress, maximum strain, and Young’s modulus were calculated. Young’s modulus was estimated from the slope of the initial linear region of the stress-strain curve.

### Statistical analysis

Statistical analyses were conducted for LC-MS, qPCR, and tensile strength measurements to assess significant differences and trends across samples. LC-MS intensity values from four biological replicates of cocoon and mucus samples were analyzed in Perseus v1.6.15.0^15^. Data were log₂-transformed and filtered to retain entries with at least three valid values. Missing values were imputed, and proteins enriched in either the cocoon or the mucus were identified using a two-sample Student’s *t*-test (p < 0.05).

Gene expression was quantified by qPCR, with expression levels normalized to the *GAPDH* transcript and calculated using the 2^-ΔCt^ method. Statistical significance was assessed using the Kruskal-Wallis rank sum test, followed by pairwise Wilcoxon tests^50,51^. Calculations were performed in R 4.4.1 with RStudio (2024.12.1 + 563).

For tensile strength measurements, data were analyzed in R version (v. 4.3.2). Data from n = 10 individuals per silk type were tested for normality and homogeneity using the Shapiro–Wilk test, and all groups showed no significant deviation from normality (p > 0.05^52^. Homogeneity of variances was confirmed using Brown & Forsythe (Levene-type) test, p > 0.05^53^. Differences in maximum stress among silk types were analyzed using a one-way ANOVA. Significant overall effects were followed by Tukey’s Honest Significant Difference (HSD) post hoc test to determine pairwise differences between species^54^.

### Reporting summary

Further information on research design is available in the Nature Portfolio Reporting Summary linked to this article.

## Supplementary information

Supplementary Information

Description of Additional Supplementary Files

Supplementary Data 1

Supplementary Data 2

Supplementary Movie 1

Reporting Summary

Transparent Peer Review file

## Source data

Source Data

## Data Availability

All the data generated in this study have been deposited in the ZENODO database [https://zenodo.org/records/17989158]. The transcriptome data were deposited in the European Nucleotide Archive (ENA) at EMBL-EBI database with the following details: Accession: ERR16052933, Study: ERP186867, Sample: ERS28374073, Experiment: ERX15443361, Submission date: 21 Dec 2025 [https://www.ebi.ac.uk/ena/browser/view/ERP186867]. The mass spectrometry proteomics data have been deposited in the ProteomeXchange Consortium via the PRIDE partner repository with the dataset identifier PXD073143. All illustrations were created by the authors and do not contain any third-party material. Source data are provided with this paper.
