# Supplementary material for: Characterization of silk-like materials from larval salivary glands of the dipteran, Keroplatus testaceus
Source: Nat Commun. 2026 Oct 5;17:11363. doi: 10.1038/s41467-026-78291-5 (PMC13728221; doi:10.1038/s41467-026-78291-5)
Supplement: Supplementary file 1 [file 41467_2026_78291_MOESM1_ESM.pdf]

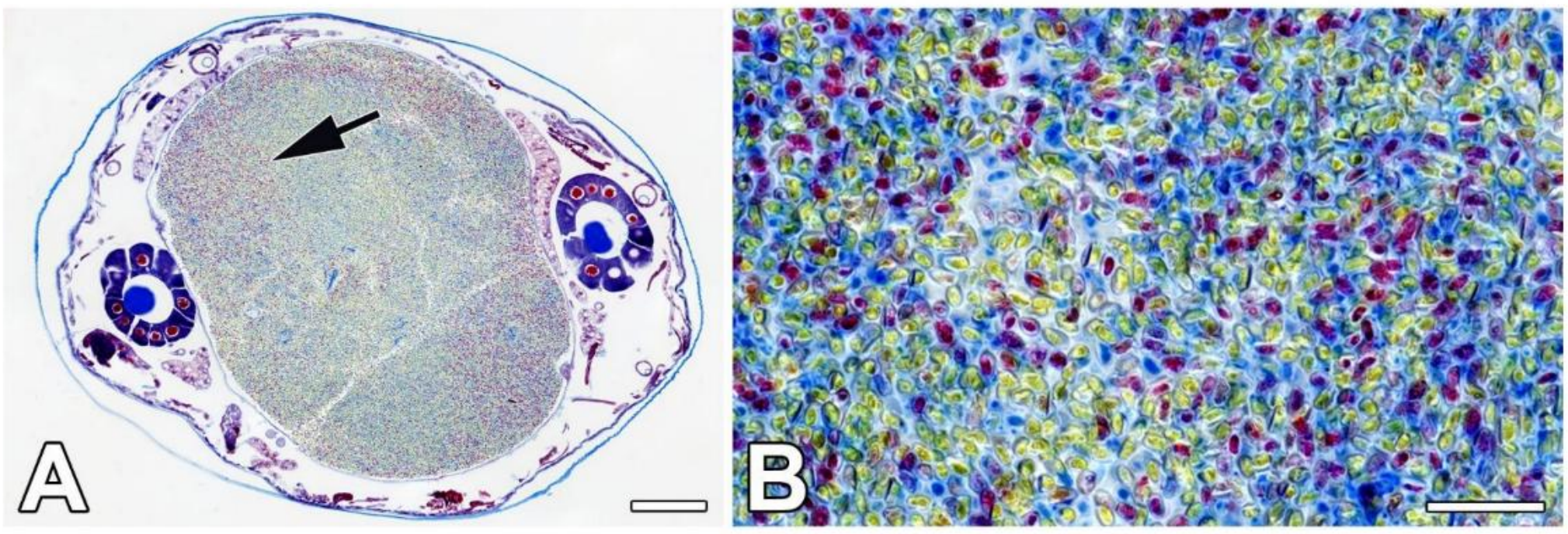

Supplementary Figure 1. Fungal spores present in the intestine of *K. testaceus* larva. (A) Cross-section of a larva showing the intestinal lumen (arrow) filled with fungal spores. (B) Higher magnification of the indicated area in panel A, illustrating the dense accumulation of fungal spores within the gut content. Scale bars: (A) 100 μm; (B) 20 μm.

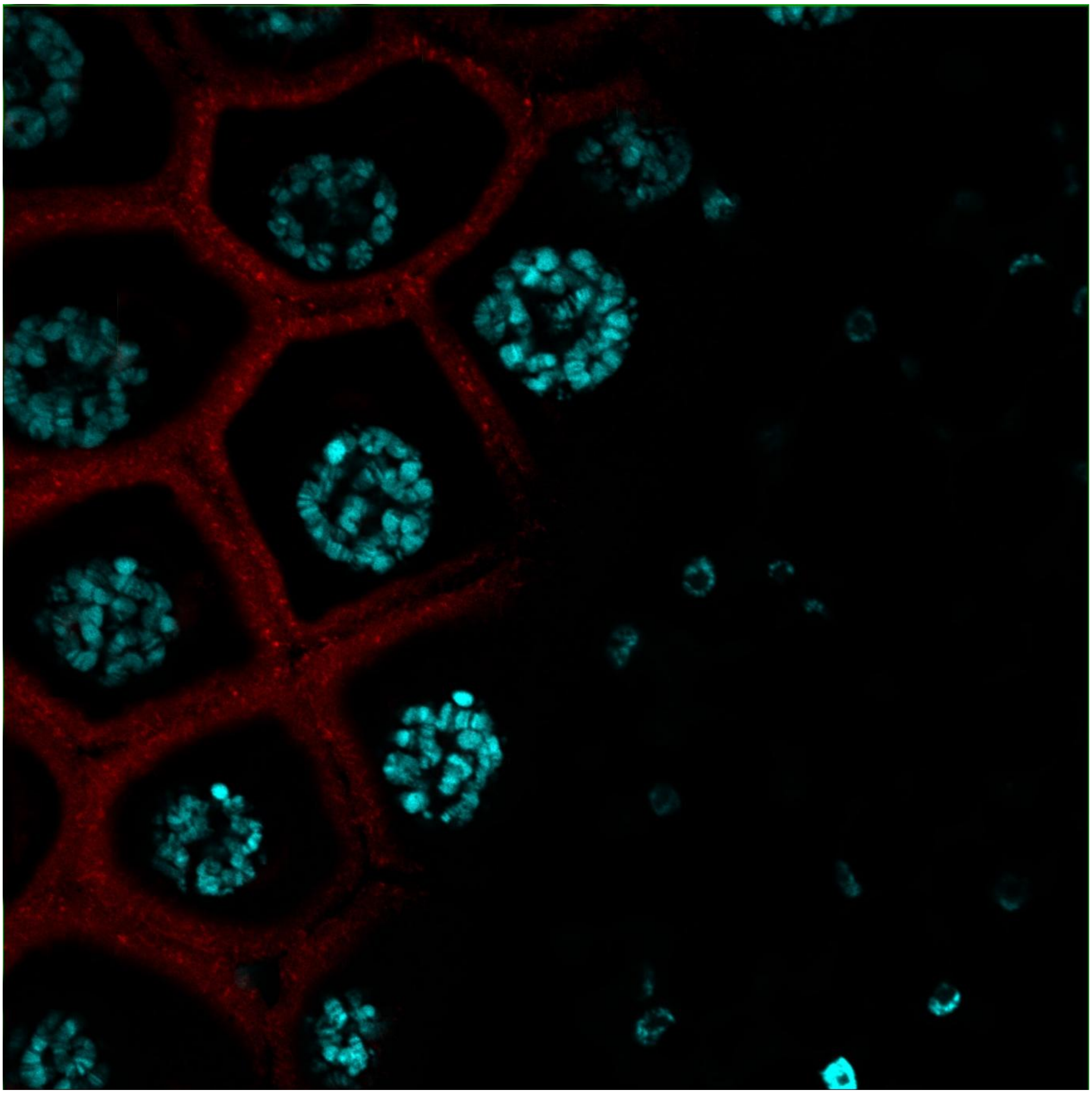

Supplementary Figure 2. Confocal microscopy of the salivary gland epithelium. Showing a single optical section stained with phalloidin to visualize actin (red). In contrast to the diffuse signal observed in the z-stack projection presented in the main figure, the single optical section clearly resolves the actin cortex surrounding the gland cells. This control image demonstrates that the blurred appearance of the actin cortex in the projection results from the overlay of multiple optical sections rather than from the underlying actin organization. Scale bar: 20 µm.

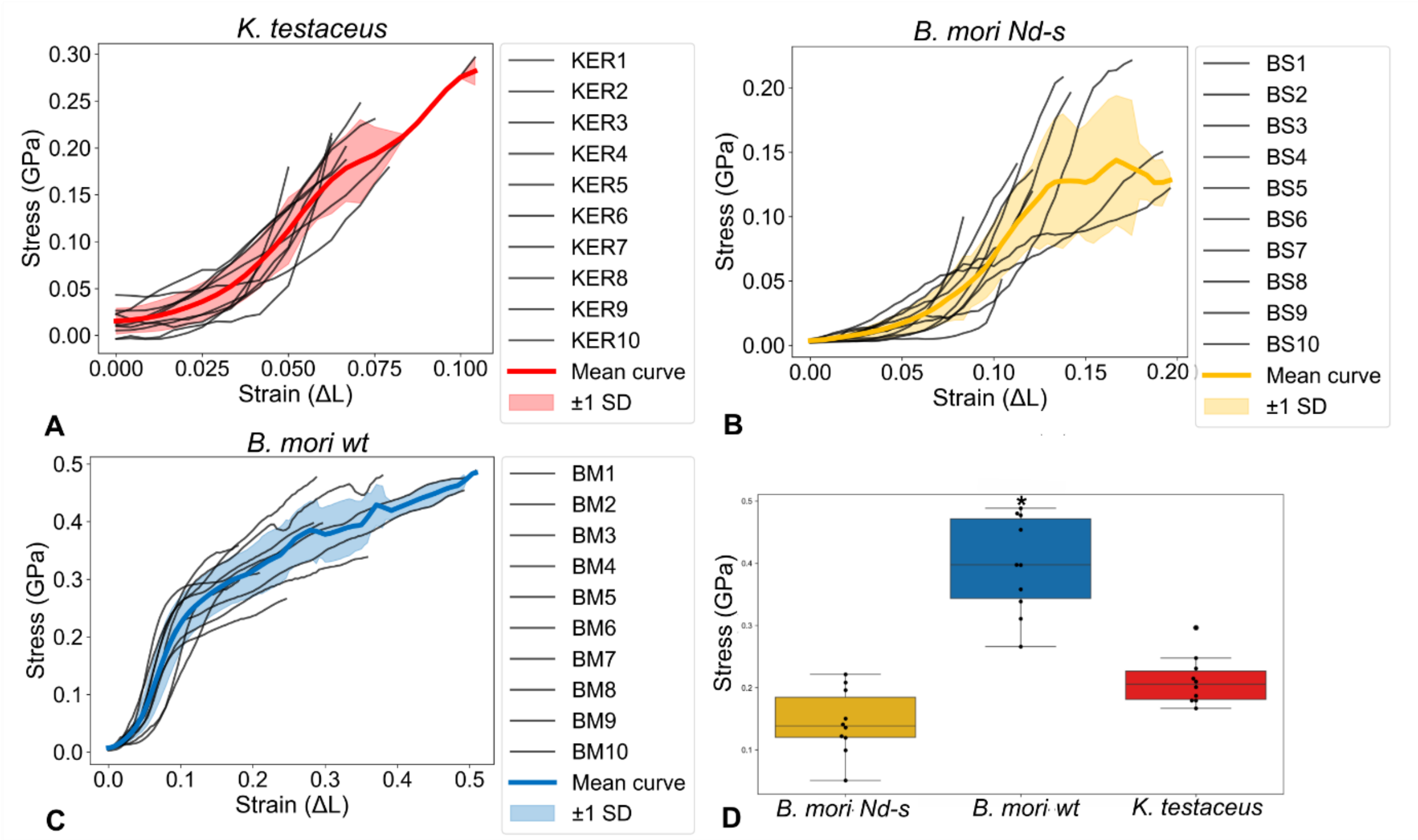

Supplementary Figure 3. Mechanical characterization of silk fibers from *K. testaceus* and *B. mori*. (A-C) Representation of stress-strain plots for the individual fibers (black lines, n=10), mean curves (colored lines), and standard deviation (shaded areas) is shown for the indicated silk type. (D) Boxplot comparison of maximum stress values across the three silk types. Each dot represents a single measurement. Statistical differences were assessed using one-way ANOVA ($F(2.\ 27) = 49.69$, $p < 0.0001$), followed by Tukey's HSD post hoc test. All pairwise comparisons were statistically significant ($p < 0.05$).

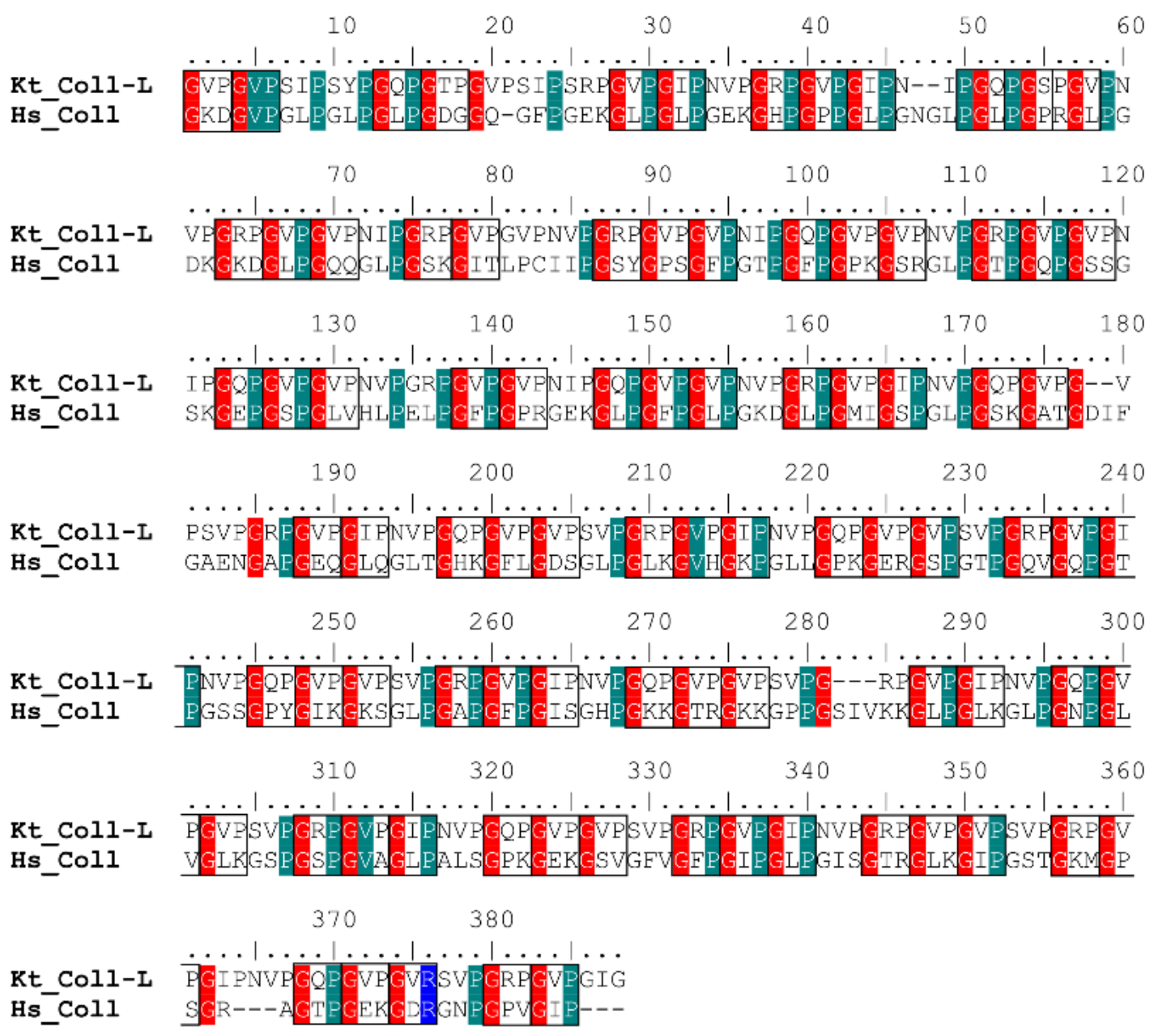

Supplementary Figure 4. Sequence alignment of the *K. testaceus* collagen-like protein (Kt_Coll-L) and *Homo sapiens* collagen I (Hs_CoII; GenBank: BAA04809.1), showing the repetitive G–X–X′ motif (black boxes) typical of collagen triple helices, where G represents glycine, X is any amino acid, and X′ is often proline (P). The collagen-like domain of *K. testaceus* shares about 40% sequence identity with *H. sapiens* collagen I (BioEdit, BLOSUM62).

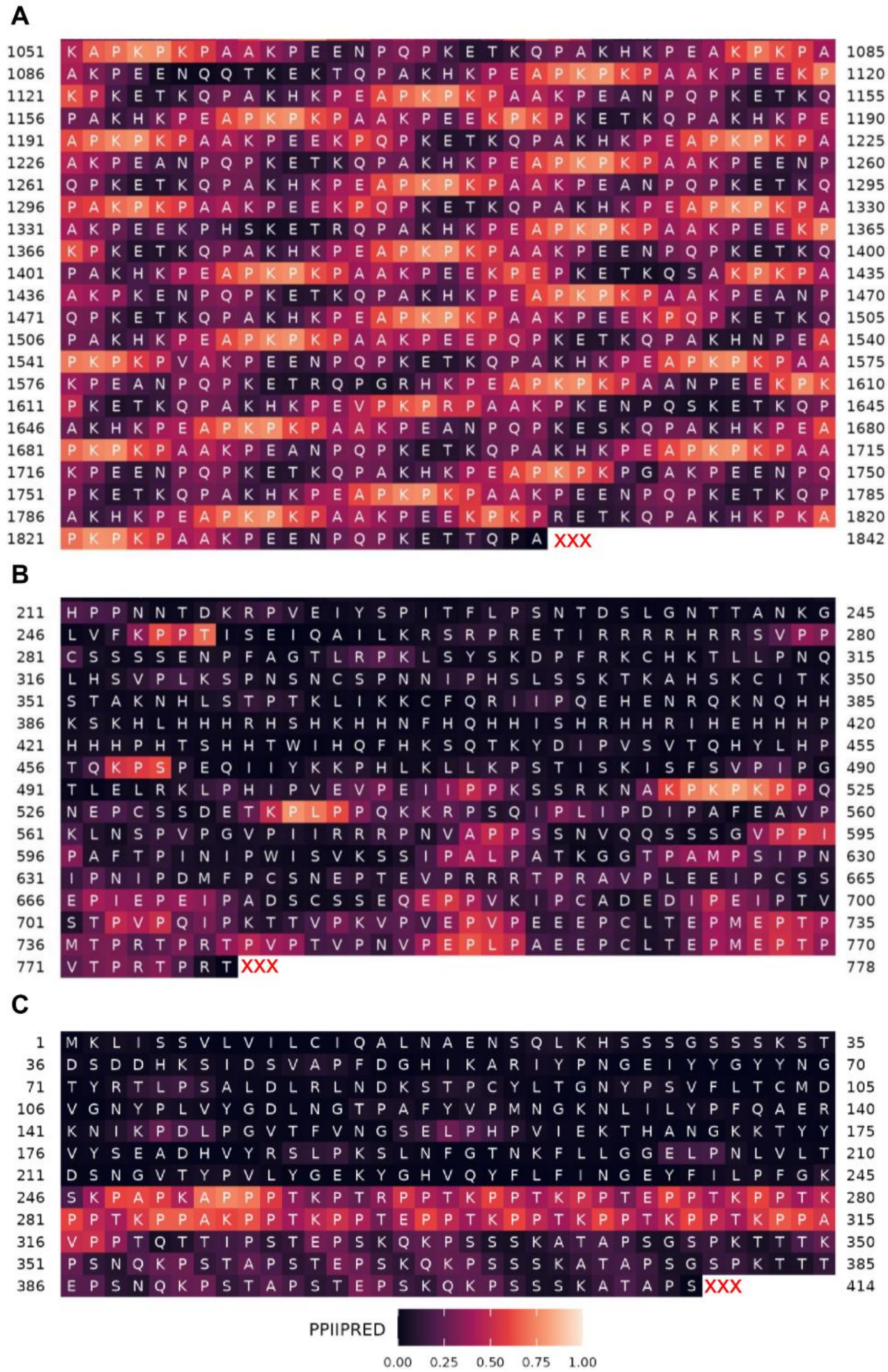

Supplementary Figure 5. Predicted PPII helices in selected proteins generated using PPIIPred [28]. (A) PK-rich protein shows predicted PPII helices across most of its sequence; only a central region is shown. (B) Mucin-like protein displays PPII helices starting in the central part of the sequence; the N-terminal region without predicted PPII helices is not shown. (C) Prolinin 8 contains a predicted PPII helix in its central region. All three proteins lack complete C-terminal regions (indicated as XXX).

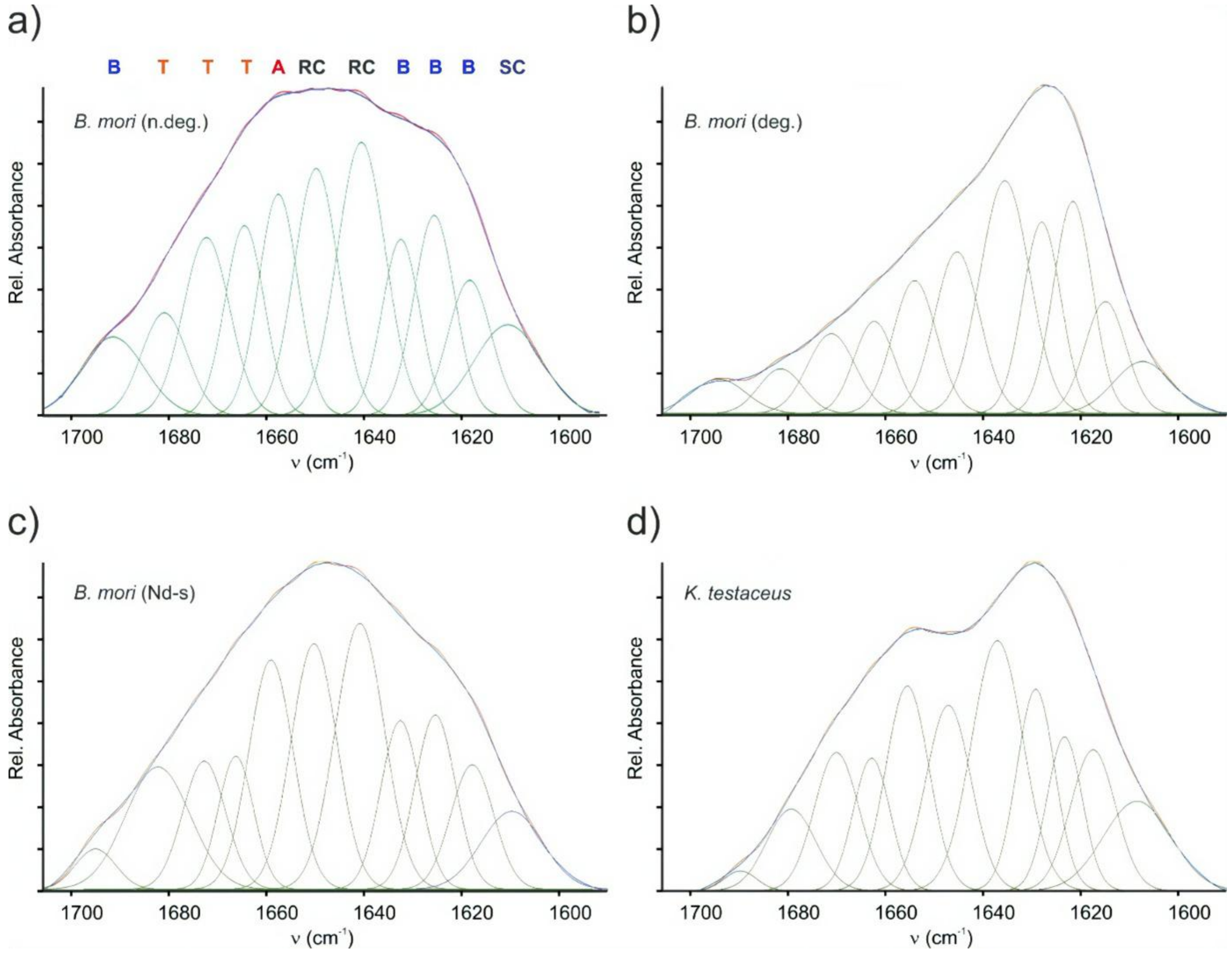

Supplementary Figure 6. Deconvolution of the amide I region of FSD-FTIR spectra. Gaussian peak fitting of the amide I band (1700–1600 cm⁻¹) for (a) *B. mori* (non-degummed), (b) *B. mori* (degummed), (c) *B. mori* (Nd-s), and (d) *K. testaceus*. Experimental spectra (blue lines) and cumulative fitted curves (red lines) are shown together with individual Gaussian components corresponding to β-sheet (B), random coil (RC), α-helix (A), β-turn (T), and side-chain contributions (SC). The secondary structure elements were assigned as follows: side chains (SC): 1595–1615 cm^{-1}, β-sheets (BS): 1616–1637 cm^{-1}, 1697–1703 cm^{-1}, random coils (RC): 1638–1655 cm^{-1}, α-helices (AH): 1656–1662 cm^{-1}, turns (T): 1663–1696 cm^{-1}. The close agreement between experimental and fitted profiles indicates reliable secondary-structure resolution within the amide I region.

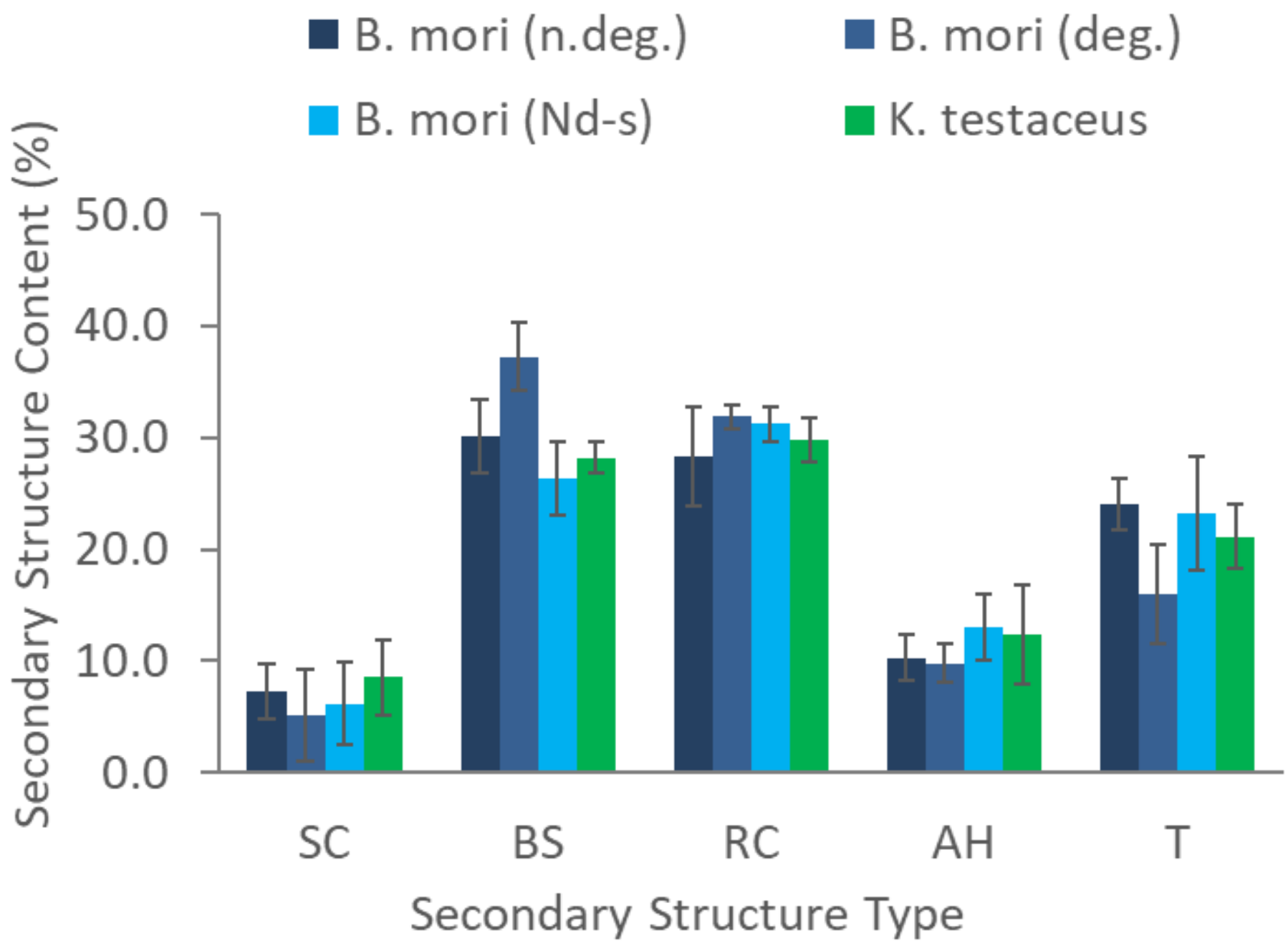

Supplementary Figure 7. Percentages of secondary structure elements in silk samples. Using the FSD-analyses Supplementary Figure 6., the content of secondary structures elements was calculated. Mean values of three to four replicates are shown.

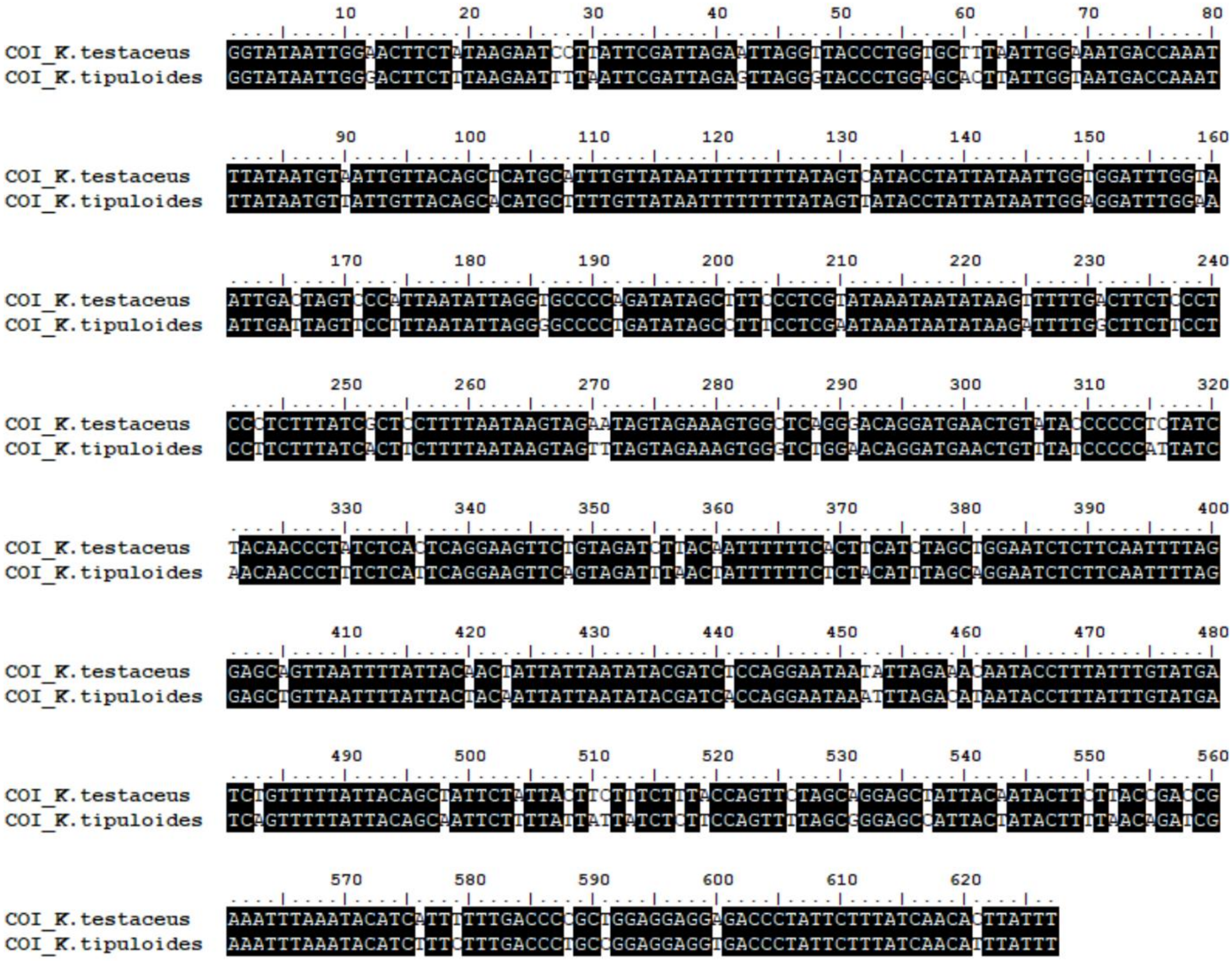

Supplementary Figure 8. Pairwise nucleotide sequence alignment of the mitochondrial COI gene of two Keroplatus species occurring in the Czech Republic, *Keroplatus testaceus* (Kt) and *Keroplatus tipuloides* (Kti). Kt_COI sequence obtained from our transcriptome, GenBank accession (PV206836.1) and Kti_COI sequence retrieved from GenBank (MT446888.1). The both sequences share 86% nucleotide sequence identity, indicating substantial divergence between the two species (BioEdit, BLOSUM62).

Species	Young's Modulus (GPa)	Yield Stress (GPa)	Max. Stress (GPa)	Max. Strain (mm)	Total Toughness (MJ/m³)
K. testaceus	**3.89 ± 0.90**	0.15 ± 0.10	0.28 ± 0.06	0.08 ± 0.01	8.01 ± 3.53
B. mori wt	**3.55 ± 0.90**	0.33 ± 0.07	0.40 ± 0.08	0.33 ± 0.11	92.07 ± 47.35
B. mori Nd-s	**0.68 ± 0.28**	0.10 ± 0.07	0.17 ± 0.04	0.15 ± 0.04	8.63 ± 4.28

Supplementary Table 1. Mechanical properties of silk fibers from different species. Values represent the mean ± standard deviation from 10 independent measurements. Reported parameters include Young's modulus, yield stress, maximum stress, maximum strain, and total toughness for *K. testaceus* and *B. mori* wt and *B. mori Nd-s* silk fibers.

Symbol	GeneBank ID	No. AA	MW	1st AA(%)	2nd AA(%)	3th AA(%)	pI	GRAVY	Cocoon intensities	Mucus intensities	p-value	Secondary structures α(%)	β(%)	dis(%)	Family
Kt_Srp1	PX584754	inc.	68	P (14.5)	S (12.3)	I (11.5)	4.22	0.019	30.10	27.44	0.11393405	20.0	17.8	62.2	Srps
Kt_Srp3A	PX584756	413	45	A (10.2)	S,L (9.4)	N.Q (7,3)	4.86	0.063	**27.20***	21.57	**0.0238074**	27.4	28.8	43.8	Srps
Kt_Srp4	PX584758	429	47	S (11.0)	I (10.7)	L (9.6)	4.85	0.147	**25.20***	21.09	**0.0120661**	28.0	26.6	45.5	Srps
Kt_Srp3B	PX584757	411	45	S (10.9)	A (10.7)	L (8.8)	4.45	0.073	24.20	20.88	0.14480591	28.7	27.7	43.6	Srps
Kt_Srp2	PX584755	660	73	S (11.1)	P (9.8)	T (8.9)	9.61	-0.427	23.80	23.09	0.75224792	21.4	17.7	60.9	Srps
Kt_Srp5	PX584759	452	52	L (10.0)	I (9.1)	T (8.0)	8.98	-0.307	21.70	23.44	0.58082758	29.2	25.7	45.1	Srps
Kt_Srp6	PX584760	418	46	L (11.5)	S (8.6)	T (7.7)	5.65	-0.020	21.00	25.22	0.16630214	31.6	30.4	38.0	Srps
Kt_Srp7	PX584761	435	47	S (11.0)	L (9.4)	A (8.0)	5.01	0.061	**26.90***	21.79	**0.03411743**	25.9	25.9	48.2	Srps
Kt_Sap1	PX584747	717	81	S (10.3)	C (9.1)	A (8.2)	5.00	-0.006	29.30	25.09	0.16806699	n.d.	n.d.	n.d.	SAPA
Kt_Sap2	PX584748	1812	209	P (11.1)	C (9.1)	N (8.5)	6.68	-0.606	25.60	22.57	0.17187182	n.d.	n.d.	n.d.	SAPA
Kt_Pxd1	PX584743	692	79	L (9.4)	I (8.7)	N (7.5)	6.09	-0.311	**28.20***	22.79	**0.01297588**	n.d.	n.d.	n.d.	Pxds
Kt_Pxd2	PX584744	304	34	L (8.9)	S (8.2)	N,I (7.6)	7.01	-0.240	**26.20***	20.26	**0.01166636**	n.d.	n.d.	n.d.	Pxds
Kt_Pxd3	PX584745	690	79	L (10.1)	N (8.4)	I (7.2)	5.47	-0.290	23.10	20.49	0.18343596	n.d.	n.d.	n.d.	Pxds
Kt_Pxd4	PX584746	691	78	L (10.4)	S (7.2)	A (6.8)	5.07	-0.238	23.00	27.02	0.11020339	n.d.	n.d.	n.d.	Pxds
Kt_Kaz1A	PX584722	268	29	P (19.0)	S (11.9)	C (7.8)	8.18	-0.462	**25.80***	20.46	**0.00985303**	11.2	15.7	73.1	PIs/ABPs
Kt_Kaz1B	PX584723	336	36	P (19.0)	S (9.5)	C (8.3)	8.71	-0.440	23.80	21.04	0.21361782	n.d.	n.d.	n.d.	PIs/ABPs
Kt_AntBac-L1	PX584698	93	10	L,V (9.7)	G (8.6)	S,P,C (7.5)	5.07	0.196	24.20	21.22	0.10489594	n.d.	n.d.	n.d.	PIs/ABPs
Kt_AntBac-L2	PX584699	137	14	P (19.7)	S (10.2)	I (9.5)	8.93	0.007	21.50	21.45	0.99756191	n.d.	n.d.	n.d.	PIs/ABPs
Kt_Sphester1	PX584753	640	74	L (8.4)	N (7.7)	A (7.5)	8.14	-0.462	24.50	21.78	0.2858091	n.d.	n.d.	n.d.	PhoEst
Kt_Metase1	PX584731	566	65	L (9.4)	N (8.0)	I (7.1)	5.95	-0.332	22.50	20.36	0.14968308	n.d.	n.d.	n.d.	PhoEst
Kt_Sepro1	PX584749	507	57	S (8.7)	G (8.1)	L (7.3)	5.45	-0.216	23.20	21.49	0.36285956	n.d.	n.d.	n.d.	SePros
Kt_Sepro2	PX584750	462	53	N,T (9.3)	L (8.7)	I,S (8.2)	7.13	-0.225	21.20	22.59	0.38572045	n.d.	n.d.	n.d.	SePros
Kt_Sepro3	PX584751	641	69	G (10.3)	L (8.6)	S (8.4)	5.74	-0.139	20.40	21.83	0.25272543	n.d.	n.d.	n.d.	SePros
Kt_ChitBP1	PX584713	629	68	T (19.6)	P (17.2)	K (7.2)	7.10	-0.702	23.40	20.47	0.1341491	2.4	16.9	80.8	Chitin BP
Kt_ChitBP2	PX584714	399	43	T (19.0)	P (18.0)	S (7.8)	6.13	-0.957	20.80	21.60	0.63431687	5.5	10.5	84.0	Chitin BP
Kt_ChitBP3	PX584714	174	19	A (9.8)	S (9.2)	T,L (8.6)	5.40	0.195	**25.60***	21.15	**0.03454068**	24.1	12.1	63.8	Chitin BP
Kt_ChitBP4	PX584716	320	34	T (13.4)	S (10.0)	A (9.4)	7.27	0.008	21.90	21.81	0.95086059	0	33.4	66.9	Chitin BP
Kt_vWC1	PX584764	312	34	C (10.3)	P (8.0)	L (7.4)	8.00	-0.057	25.40	23.36	0.38546024	n.d.	n.d.	n.d.	vWC
Kt_vWC2	PX584765	297	33	C (11.1)	S,N (7.4)	P (7.1)	7.21	-0.196	23.70	21.72	0.09725888	n.d.	n.d.	n.d.	vWC
Kt_Muc-L1	PX584732	inc.	86	P (15.9)	S (10.0)	T,K (7.7)	9.75	-0.707	**33.90***	22.04	**0.00013099**	6.6	0	93.4	Mucins
Kt_Muc-L2	PX584680	300	31	T (28.00)	P (11.7)	A (10.7)	10.12	-0.599	21.80	23.06	0.4815694	22.3	0	77.7	Mucins
Kt_Coll-L	PX584703	inc.	92	P (26.9)	G (21.6)	V (10.7)	11.4	-0.474	26.30	23.12	0.11791825	0	0.4	99.6	orphan
Kt_Ser-L	PX584684	515	50	S (23.7)	A (14.8)	Q (12.4)	4.22	-0.143	26.10	27.40	0.57223632	49.9	0	50.1	orphan
Kt_Zon-L1	PX584692	327	35	A (12.2)	C (11.3)	N (9.2)	8.32	-0.251	**27.90***	22.69	**0.01675995**	80.3	0	19.7	orphan
Kt_Metase2	PX584679	940	109	L (9.6)	S (7.7)	E (6.8)	5.77	-0.424	**26.00***	21.12	**0.02304132**	45.2	18.5	36.3	orphan
Kt_SSP	PX584686	165	18	P (11.5)	V (9.7)	G,Y,I (7.9)	9.39	-0.132	24.10	23.03	0.67138676	n.d.	n.d.	n.d.	orphan
Kt_Gluase	PX584669	523	60	I (7.8)	N (7.3)	L (6.9)	5.59	-0.332	23.40	25.57	0.40069208	n.d.	n.d.	n.d.	orphan
Kt_Hemcyn	PX584671	712	84	L (9.8)	Y (8.6)	V (7.9)	5.79	-0.364	22.90	22.08	0.73340009	n.d.	n.d.	n.d.	orphan
Kt_VasEGF	PX584690	292	33	N,D,K (7.9)	V (7.5)	L,P (7.2)	7.45	-0.543	22.70	21.65	0.41085662	n.d.	n.d.	n.d.	orphan
Kt_GlyHyd1	PX584670	647	74	L (9.9)	G (7.7)	I (7.1)	5.41	-0.335	22.50	21.33	0.59748527	n.d.	n.d.	n.d.	orphan
Kt_Ven-L	PX584672	657	73	K (9.7)	E (8.8)	V (8.2)	5.17	-0.496	21.90	24.74	0.15442289	n.d.	n.d.	n.d.	orphan
Kt_AntBac-L3	PX584667	139	14	A (18.7)	N,P (9.4)	V,L,K (7.9)	9.35	-0.261	21.80	22.25	0.8033872	n.d.	n.d.	n.d.	orphan
Kt_HisPho	PX584772	531	63	L (10.2)	K (8.1)	F (7.3)	8.34	-0.517	21.30	20.86	0.83585654	n.d.	n.d.	n.d.	orphan
Kt_Apo1	PX584768	201	22	S (10.4)	L (9.5)	T,V (9.0)	4.50	0.033	20.90	22.27	0.52498611	n.d.	n.d.	n.d.	orphan
Kt_Apo2	PX584769	251	28	L (10.4)	T (8.4)	S,Y (7.2)	5.22	-0.253	20.80	21.98	0.49103711	n.d.	n.d.	n.d.	orphan
Kt_TrypSP	PX584783	283	30	G (11.3)	V (8.8)	A (8.5)	9.10	0.104	20.60	22.76	0.20670486	n.d.	n.d.	n.d.	orphan
Kt_Iso1	PX584776	202	22	G (13.4)	V (9.4)	A (8.9)	6.85	-0.066	20.30	**24.78***	**0.0131539**	11.4	26.7	61.9	orphan
Kt_PDI	PX584779	496	56	K (10.1)	D,E (9.1)	L (8.9)	4.80	-0.354	20.20	20.74	0.60103113	n.d.	n.d.	n.d.	orphan
Kt_Fer1	PX584771	248	28	L (9.7)	S (8.9)	A (8.5)	5.72	-0.431	19.10	**22.37***	**0.00807227**	n.d.	n.d.	n.d.	orphan

Supplementary Table 2A. List of 49 candidate silk proteins with known homologs identified in *K. testaceus*, with their biochemical characteristics. **Abbreviations:** No. AA – number of amino acids; MW – molecular weight; AA – amino acid composition; pI – isoelectric point; GRAVY – grand average of hydropathy; C – cocoon; M – mucus. Protein families: Srps – serpins; SAPA – saposins; Pxds – peroxidases; PIs/ABPs – protease inhibitors and antibacterial peptides; PhoEst – phosphoesterases; SePros – serine proteases; Chitin BP – chitin-binding proteins; vWC – von Willebrand factor type C domain; inc. – incomplete properties calculated from partial sequences; α-alpha helices; β-beta strands; dis-disordered regions; n.d. - not determined. Orphans - individual proteins that do not belong to any protein family and show no detectable homology to each other. Protein intensities were $\log_2$-transformed for statistical analyses. Exact p-values are provided in the table (two-sided t-test). Asterisks indicate proteins significantly enriched in the respective silk material ($p < 0.05$).

Protein name	Symbol	GeneBank ID	No. AA	MW	1st AA(%)	2nd AA(%)	3th AA(%)	pI	GRAVY	Cocoon intensities	Cocoon intensities	p-values	Secondary structures			Family
													α(%)	β(%)	dis(%)	
Prolinin 2	Kt_Plin2	PX584736	inc.	79	P (18.1)	I (11.1)	S (9.3)	5.64	-0.270	**33.30***	22.51	**0.00053166**	2.9	0	97.1	Plins
Prolinin 1	Kt_Plin1	PX584735	inc.	102	P (12.6)	K (9.0)	L (8.8)	7.26	-0.497	29.80	29.87	0.973690427	5.8	30.8	63.3	Plins
Prolinin 4	Kt_Plin4	PX584738	656	73	P (10.4)	S (9.9)	G (8.8)	9.18	-0.229	26.60	25.15	0.559939983	0.5	43.0	56.6	Plins
Prolinin 5	Kt_Plin5	PX584739	835	85	P (17.7)	G (11.1)	S (10.7)	9.47	-0.338	26.40	24.98	0.47313993	2.2	21.2	76.6	Plins
Prolinin 6	Kt_Plin6	PX584740	760	81	P (15.1)	S (10.9)	G (8.3)	8.94	-0.375	26.10	24.24	0.279116605	2.5	36.3	61.2	Plins
Prolinin 8	Kt_Plin8	PX584742	inc.	45	P (17.1)	S (11.1)	K,T (10.6)	9.63	-0.782	23.50	23.00	0.844871893	1.5	19.3	79.2	Plins
Prolinin 7	Kt_Plin7	PX584741	inc.	66	P (15.1)	S (10.8)	Q (10.2)	9.78	-0.992	22.90	20.47	0.144268947	5.1	25.1	69.8	Plins
Prolinin 3	Kt_Plin3	PX584737	560	66	P (17.0)	K (11.1)	H (8.8)	9.78	-1.091	21.80	22.76	0.744676774	0	14.3	85.7	Plins
SLrich protein	Kt_SLRP	PX584752	242	26	S (12.4)	L (10.3)	G,Y (8.7)	8.41	-0.072	**28.70***	23.88	**0.038964844**	2.5	35.1	62.4	LXL motif
YL rich protein	Kt_YLRP	PX584763	230	26	Y (11.3)	L (10.9)	G (9.1)	9.25	-0.050	25.80	24.31	0.623555351	n.d.	n.d.	n.d.	LXL motif
PK rich protein	Kt_PKRP	PX584734	inc.	204	P (26.7)	K (25.9)	A (13.8)	10.00	-2.082	**29.20***	20.98	**0.007149861**	2.3	0	97.7	HHP
EQA rich protein	Kt_EQARP	PX584709	346	40	E (16.8)	Q (14.5)	A (13.3)	8.74	-1.560	23.00	**28.49***	**0.004732465**	52.6	0	47.4	HHP
EK rich protein	Kt_EKRP	PX584708	560	65	E (20.7)	K (18.4)	A (10.9)	6.82	-2.016	22.20	**28.50***	**0.005617759**	40.7	0	59.3	HHP
IA rich protein	Kt_IARP	PX584717	307	33	I (11.1)	A (10.7)	N (10.4)	5.47	0.176	**30.40***	20.90	**0.002988781**	76.2	4.6	19.2	ARP
A rich protein 1A	Kt_ARP1A	PX584700	282	30	A (16.3)	L (12.1)	S,N (8.2)	5.08	0.258	27.20	25.53	0.373997994	n.d.	n.d.	n.d.	ARP
A rich protein 1B	Kt_ARP1B	PX584701	284	30	A (12.8)	L (10.6)	N (10.2)	9.08	0.159	24.80	29.21	0.075558755	n.d.	n.d.	n.d.	ARP
LNI rich protein	Kt_LNIRP	PX584727	279	30	L (12.2)	N (10.4)	I,V (9.3)	4.92	0.239	24.70	21.18	0.045318743	n.d.	n.d.	n.d.	ARP
A rich protein 1C	Kt_ARP1C	PX584702	283	29	A (19.4)	L (13.4)	K (8.5)	7.66	0.232	23.70	24.39	0.78597832	n.d.	n.d.	n.d.	ARP
ANL rich protein	Kt_ANLRP	PX584697	285	31	A (13.0)	N (12.3)	L (11.2)	7.71	0.244	21.20	23.55	0.302292266	n.d.	n.d.	n.d.	ARP
L rich protein 1	Kt_LRP1	PX584729	309	33	L (14.2)	I (10.7)	A (9.7)	4.78	0.383	**29.40***	21.17	**0.007812585**	64.1	0	35.9	LRP
L rich protein 2	Kt_LRP2	PX584730	290	32	L (10.7)	I (9.7)	A,V (9.3)	4.97	0.187	**27.30***	22.21	**0.017153464**	80.3	0	19.7	LRP
IS rich protein	Kt_ISRP	PX584720	283	31	I (14.8)	S (11.0)	L (9.9)	5.73	0.211	27.70	22.76	0.052108559	n.d.	n.d.	n.d.	LIS
LIS rich protein	Kt_LISRP	PX584726	344	37	L (11.9)	I (11.6)	S (11.3)	6.74	0.154	25.40	20.66	0.201269398	n.d.	n.d.	n.d.	LIS
LI rich protein 1	Kt_LIRP1	PX584724	297	33	I (12.5)	L (10.4)	S (8.1)	4.95	0.243	20.80	24.02	0.116602873	n.d.	n.d.	n.d.	LIS
LI rich protein 2	Kt_LIRP2	PX584725	361	40	L (11.1)	I (10.0)	A (9.4)	8.96	0.070	19.80	**23.27***	**0.028719729**	n.d.	n.d.	n.d.	LIS
ILS rich protein	Kt_ILSRP1	PX584719	294	33	I (10.9)	L(10.2)	S(8.8)	6.12	0.174	24.20	21.37	0.215751289	n.d.	n.d.	n.d.	LIS
ANGL rich protein	Kt_ANGLRP	PX584696	366	37	A (12.0)	N (10.9)	L (10.7)	4.87	0.258	23.50	23.94	0.888835429	n.d.	n.d.	n.d.	DUF
AIS rich protein	Kt_AISRP	PX584693	303	32	A (11.6)	S (10.9)	I (10.2)	4.98	0.345	20.00	21.43	0.372500056	n.d.	n.d.	n.d.	DUF
AL rich protein 1	Kt_ALRP1	PX584694	282	30	A (13.5)	L (12.8)	N,I,S (8.9)	4.59	0.260	25.70	21.80	0.066520168	n.d.	n.d.	n.d.	HydBic P30
LN rich protein	Kt_LNRP	PX584728	283	31	L (13.1)	I (10.6)	N (10.2)	5.95	0.164	24.20	20.66	0.250846441	n.d.	n.d.	n.d.	HydBic P30
IL rich protein	Kt_ILRP	PX584718	299	34	L (10.7)	I (10.4)	N (8.0)	5.97	0.058	23.50	23.49	0.986984867	n.d.	n.d.	n.d.	HydBic P30
AL rich protein 2	Kt_ALRP2	PX584695	290	31	A (12.8)	L (11.0)	I (9.7)	5.01	0.283	22.30	24.82	0.309344431	n.d.	n.d.	n.d.	HydBic P30
VS rich protein	Kt_VSRP	PX584762	179	19	V (12.8)	S (11.2)	N (8.9)	6.70	-0.131	24.90	22.04	0.20312059	n.d.	n.d.	n.d.	PGSPV motif
NS rich protein	Kt_NSRP	PX584733	205	22	N (12.2)	S (10.7)	I (9.3)	5.66	-0.051	24.40	22.47	0.368081037	n.d.	n.d.	n.d.	PGSPV motif
IT rich protein	Kt_ITRP	PX584721	151	16	I (13.2)	T (11.9)	N (9.3)	8.19	0.034	24.10	20.84	0.048795376	n.d.	n.d.	n.d.	PGSPV motif
11kDa, hydrophobic protein A	Kt_Hybic11A	PX584710	113	11	A,L (16.8)	G (13.3)	V (8.0)	6.22	0.544	**24.50***	20.71	**0.001896845**	n.d.	n.d.	n.d.	SAP
11kDa, hydrophobic protein B	Kt_Hybic11B	PX584711	116	11	A (16.4)	G (13.8)	L (12.1)	4.58	0.483	**24.00***	20.86	**0.02459969**	n.d.	n.d.	n.d.	SAP
12kDa, hydrophobic protein	Kt_Hybic12	PX584712	123	12	A (26.8)	T (8.9)	V (8.1)	4.61	0.498	22.80	25.16	0.04404075	n.d.	n.d.	n.d.	SAP
CW protein 1	Kt_CWP1	PX584704	294	33	K (11.2)	A (9.9)	L (8.8)	9.09	-0.369	24.10	25.60	0.320538113	n.d.	n.d.	n.d.	HydLic P30
CW protein 2A	Kt_CWP2A	PX584705	275	32	L (11.6)	S (8.4)	I,N (7.6)	8.99	-0.155	23.00	**28.05***	**0.017044941**	68.7	2.9	28.4	HydLic P30
CW protein 2B	Kt_CWP2B	PX584706	275	32	L(12.4)	S (8.4)	N (7.6)	5.89	-0.230	22.70	**26.82***	**0.033746265**	n.d.	n.d.	n.d.	HydLic P30
CW protein 3	Kt_CWP3	PX584707	267	30	L (11.6)	N (10.5)	S (9.7)	6.33	-0.206	19.60	**25.30***	**0.008510003**	78.7	0	21.3	HydLic P30
KPDA tail protein	Kt_KPDA	PX584673	414	41	A (27.8)	T (12.6)	P (11.8)	7.62	-0.571	**28.30***	22.80	**0.012975876**	7.7	7.0	85.3	orphan
LN rich protein 11kDa	Kt_LNRP11	PX584676	104	11	L (14.4)	N (12.5)	T (8.7)	6.25	-0.018	**27.90***	20.55	**0.00115509**	31.7	0	68.3	orphan
TP rich protein	Kt_TPRP	PX584688	200	23	T (11.5)	P (10.0)	I (8.5)	9.75	-0.488	**26.60***	20.65	**0.012808595**	31.0	4.0	65.0	orphan
SCP protein	Kt_ SCP	PX584666	272	30	N (10.7)	I,T (7.4)	K,G (7.0)	7.54	-0.469	**25.60***	20.59	**0.019087006**	n.d.	n.d.	n.d.	orphan
IN rich protein	Kt_INRP	PX584674	313	35	N,I (9.6)	L (8.9)	T (8.3)	4.72	-0.067	25.00	20.94	0.163122938	n.d.	n.d.	n.d.	orphan
TG rich protein	Kt_TGRP	PX584687	147	15	T,G (10.2)	N (9.5)	S (8.8)	6.56	-0.402	24.40	20.77	0.143704067	n.d.	n.d.	n.d.	orphan
C tail leucine zipper	Kt_CTLZ	PX584668	285	31	A (10.9)	L,S (9.8)	I (9.5)	6.30	-0.055	23.90	22.41	0.321473715	n.d.	n.d.	n.d.	orphan
TS rich protein	Kt_TSRP	PX584689	531	58	T (17.3)	S (13.9)	N (11.7)	10.64	-0.856	23.90	20.86	0.199249792	n.d.	n.d.	n.d.	orphan
NIA rich protein	Kt_NIARP	PX584681	281	31	N (13.2)	I (11.4)	A (10.3)	6.90	-0.068	23.70	22.16	0.235619328	n.d.	n.d.	n.d.	orphan
I rich protein	Kt_IRP	PX584675	355	38	I (12.1)	K (9.6)	A (9.3)	9.45	0.241	23.70	24.63	0.765825687	n.d.	n.d.	n.d.	orphan
LS rich protein 1	Kt_LSRP1	PX584677	273	30	L (11.0)	S (10.6)	N (9.5)	4.67	-0.153	23.10	22.49	0.690588015	n.d.	n.d.	n.d.	orphan
N rich protein 1	Kt_NRP1	PX584682	165	18	N (15.8)	I (9.7)	L,T (8.5)	4.99	-0.439	22.90	20.67	0.341088936	n.d.	n.d.	n.d.	orphan
V rich protein	Kt_VRP	PX584691	259	29	A (8.9)	E,L,K (8.5)	Q (7.3)	4.95	-0.133	22.10	22.30	0.946766334	n.d.	n.d.	n.d.	orphan
LS rich protein 2	Kt_LSRP2	PX584678	397	43	L (10.8)	S (10.1)	A (9.6)	4.82	0.014	22.10	20.49	0.33959084	n.d.	n.d.	n.d.	orphan
N rich protein 2	Kt_NRP2	PX584683	290	32	N (10.7)	L (8.3)	A,V (7.9)	4.86	-0.022	21.90	20.94	0.655952063	n.d.	n.d.	n.d.	orphan
S rich protein	Kt_SRP	PX584685	inc.	17	S (18.8)	N (9.4)	T,V (8.1)	5.18	-0.554	21.70	20.70	0.433603998	n.d.	n.d.	n.d.	orphan
AL rich protein 3	Kt_ALRP3	PX584767	250	27	A (14.0)	L (10.8)	N (8.8)	5.47	0.048	21.60	24.93	0.128147241	n.d.	n.d.	n.d.	orphan
ILA rich protein 1	Kt_ILARP1	PX584773	291	32	I,L (10.3)	A (9.3)	N (8.6)	5.84	0.154	21.50	21.89	0.842312974	n.d.	n.d.	n.d.	orphan
AS rich protein	Kt_ASRP	PX584770	244	26	A (14.3)	S (11.9)	N (9.8)	8.53	0.171	21.50	21.79	0.874373077	n.d.	n.d.	n.d.	orphan
N rich protein 3	Kt_NRP3	PX584778	239	27	N (10.5)	S,V (9.6)	L (8.4)	7.63	-0.080	21.40	22.16	0.603412512	n.d.	n.d.	n.d.	orphan
AG rich short peptide 11	Kt_AGRSP11	PX584766	116	11	A (24.1)	G (14.7)	L (12.9)	4.75	0.520	21.20	21.72	0.796437741	n.d.	n.d.	n.d.	orphan
PQ rich protein	Kt_PQRP	PX584780	469	53	P (20)	Q (11.9)	S (11.7)	8.64	-1.158	20.90	21.12	0.735570979	n.d.	n.d.	n.d.	orphan
LSI rich protein	Kt_LSIRP	PX584777	300	33	L (13.0)	S (11.7)	I (10.7)	8.46	0.051	20.90	21.26	0.855361436	n.d.	n.d.	n.d.	orphan
Putative transmembrane protein	Kt_PTP	PX584781	224	22	A (25.0)	P (17.9)	L (8.5)	6.18	0.187	20.90	**24.47***	**0.032395017**	n.d.	n.d.	n.d.	orphan
ILA rich protein 2	Kt_ILARP2	PX584774	265	29	I (10.9)	L (10.6)	A,S (9.8)	4.90	0.100	20.60	23.53	0.18645551	n.d.	n.d.	n.d.	orphan
Q rich protein	Kt_QRP	PX584782	363	39	Q (12.4)	S (9.9)	A (9.4)	10.24	-0.332	20.30	**25.43***	**0.009410717**	n.d.	n.d.	n.d.	orphan
IN rich protein	Kt_INRP2	PX584775	344	39	N,I (10.2)	L (9.6)	D (7.8)	5.12	-0.269	19.30	**22.85***	**0.041467227**	n.d.	n.d.	n.d.	orphan

Supplementary Table 2B. List of 69 novel candidate silk proteins identified in *K. testaceus*, with their biochemical characteristics. Abbreviations: No. AA – number of amino acids; MW – molecular weight; AA – amino acid composition; pI – isoelectric point; GRAVY – grand average of hydropathy; C – cocoon; M – mucus. Protein families: Plins – prolinins; LXL-leucine-x-leucine motif; SAP – silk-associated proteins (similar to *Arachnocampa richardsae* (AGC26507.1)); HHP – highly hydrophilic proteins; ARP – alanine-rich proteins; LRP – leucine-rich proteins; LIS – leucine–isoleucine–serine-rich proteins; DUF – domain of unknown function; HydBic P30 – hydrophobic 30 kDa proteins; HydLic P30 – hydrophilic 30 kDa proteins; inc. – incomplete properties calculated from partial sequences, α-alpha helices; β-beta strands; dis-disordered regions; n.d. - not determined. Orphans - individual proteins that do not belong to any protein family and show no detectable homology to each other. Asterisks indicate proteins significantly enriched in the respective silk material. Orphans - individual proteins that do not belong to any protein family and show no detectable homology to each other. Protein intensities were log_2-transformed for statistical analyses. Exact p-values are provided in the table (two-sided t-test). Asterisks indicate proteins significantly enriched in the respective silk material ($p < 0.05$).

Protein name	Symbol	PFAM* domains	Domains location within protein (start to end)
von Willebrand factor 1	Kt_vWC1	1x VWC	83-154
von Willebrand factor 2	Kt_vWC2	1x VWC	71-141
Zonadhesin-like 1	Kt_Zon-L1	4x TIL	32-91; 106-165; 180-240; 255-314
Chitin-binding protein 1	Kt_ChBP1	5x ChtBD	11-76; 197-251; 289-346; 444-502; 555-612
Chitin-binding protein 2	Kt_ChBP2	2x ChtBD	32-93; 303-361
Chitin-binding protein 3	Kt_ChitBP3	1x ChtBD	91-145
Chitin-binding protein 4	Kt_ChitBP4	2x ChtBD	10-164; 211-263
Serpin 1	Kt_Srp1	1x Serpin	282-639
Serpin 2	Kt_Srp2	1x Serpin	52-657
Serpin 3A	Kt_Srp3A	1x Serpin	53-410
Serpin 3B	Kt_Srp3B	1x Serpin	51-408
Serpin 4	Kt_Srp4	1x Serpin	61-425
Serpin 5	Kt_Srp5	1x Serpin	49-452
Serpin 6	Kt_Srp6	1x Serpin	33-418
Serpin 7	Kt_Srp7	1x Serpin	68-436
Saposin 1	Kt_Sap1	4x SapA	24-53; 326-357; 369-397; 459-487
Saposin 2	Kt_Sap2	5x SapA	265-297; 948-977; 1494-1524; 1578-1607; 1662-1692
Peroxidase 1	Kt_Pxd1	1x Peroxidase	135-667
Peroxidase 2	Kt_Pxd2	1x Peroxidase	136-299
Peroxidase 3	Kt_Pxd3	1x Peroxidase	135-665
Peroxidase 4	Kt_Pxd4	1x Peroxidase	136-667
Kazal 1A	Kt_Kaz1A	2x Kazal	102-150; 186-234
Kazal 1B	Kt_Kaz1B	3x Kazal	102-150; 185-233; 266-314
Serine protease 1	Kt_Sepro1	1x Peptidase_S28	58-487
Serine protease 2	Kt_Sepro2	1x Peptidase_S28	34-451
Serine protease 3	Kt_Sepro3	1x Peptidase_S28	61-492
Trypsin-like serine protease	Kt_TrypSP	1x Tryp_SPc	43-273
Sphingomyelin phosphodiesterase 1	Kt_Sphester1	1x SapB/ 1x Metallophos	36-117/ 153-421
Metallophosphatase 1	Kt_Metase1	1x SapB, 1x Metallophos	22-103/ 139-403
Glucosylceramidase	Kt_Gluase	1x Glyco_hydro_30	114-456
Metalloaminopeptidase 2	Kt_Metase2	1x Peptidase_M1	259-502
Glyco-hydrolase 1	Kt_GlyHyd1	1x Glyco_hydro_42	61-366
Isomerase 1	Kt_Iso1	1x Pro_isomerase	29-186
Histidine phosphatase	Kt_HisPho	1x His_Phos_2	76-422

Supplementary Table 3. Overview of selected silk-related proteins identified in the SYGs of *K. testaceus* and their corresponding domain architectures. *Identified by Pfam database with SMART.
