# Supplementary material for: Characterization of silk-like materials from larval salivary glands of the dipteran, Keroplatus testaceus
Source: Nat Commun. 2026 Oct 5;17:11363. doi: 10.1038/s41467-026-78291-5 (PMC13728221; doi:10.1038/s41467-026-78291-5)
Supplement: Supplementary file 2 [file 41467_2026_78291_MOESM2_ESM.pdf]

Description of Additional Supplementary Files

File Name: Supplementary Data 1

Description: List of *K. testaceus* silk-related genes and corresponding primer sequences used for qRT-PCR analysis. The table includes the protein name, gene symbol, forward (Fw) and reverse (Rv) primer sequences, expected product size (PS) in base pairs (bp), and annealing temperature (Tm, °C). Primers were designed using Primer3Plus software [44].

File Name: Supplementary Data 2

Description: Predicted 3D structures of selected proteins. For each protein, two views are presented, rotated approximately by 180° to illustrate overall structural organization. Structures are colored by pLDDT confidence (blue = high, red = low). All protein structure predictions were generated using AlphaFold2 implemented on the Galaxy platform (tool ID: toolshed.g2.bx.psu.edu/repos/galaxy-australia/alphafold2/alphafold/2.3.2+galaxy4).

File Name: Supplementary Movie 1

Description: *K. testaceus* larva feeding on fungus.
The video shows a larva feeding on fungal material while producing mucus and consuming it together with fungal spores. The recording highlights the strength and adhesive properties of the mucus, as the larva is able to move upside down on the fungal surface against gravity.
