# Supplementary material for: Characterization of silk-like materials from larval salivary glands of the dipteran, Keroplatus testaceus
Source: Nat Commun. 2026 Oct 5;17:11363. doi: 10.1038/s41467-026-78291-5 (PMC13728221; doi:10.1038/s41467-026-78291-5)
Supplement: Supplementary file 4 [file 41467_2026_78291_MOESM7_ESM.pdf]

nature portfolio

Peer Review File

Characterization of silk-like materials from larval salivary glands of the dipteran, Keroplatus testaceus

Corresponding Author: Professor Michal Zurovec

This file contains all reviewer reports in order by version, followed by all author rebuttals in order by version.

Version 0:

Reviewer comments:

Reviewer #1

(Remarks to the Author)

The manuscript "Structural and compositional properties of silk-like materials produced by the larval salivary glands of Keroplatus testaceus (Diptera)" of Kmet et al. deals with characterization of special material from salivary glands (SGs) of the gnat fly Keroplatus that produces silk-like secretion, which subtantially differs from other dipetrans, notably model species such as Drosophila. In contrast to other insect groups, mainly Lepidoptera, silk materials from dipterans remain largely unexplored. This paper represents initial and potentially important contribution to this field as authors used variety of techniques to provide elementary information on the composition and properties of two distinct secretions of the Keroplatus SGs, the mucus web and the cocoon. Although gnats produce silk-like secretion, neither of them contains fibroin. Both of these secretions are composed of a complex mixture of hydrophilic proteins, including collagen-like, mucin-like, novel proline-rich proteins, peroxidases, protease inhibitors, and others. Authors performed structural predictions and FTIR analysis of the cocoon revealing the presence of secondary structures, including β-sheets, α-helices, and polyproline II helices. Secretion is highly hydrated, rich in proline-containing proteins, and exhibits relatively high mechanical strength.

Major points:

1. Phalloidin which is supposed to bind filamentous actin in Figure 3F is red color, but red signal is diffused over whole cytoplasm. Is this real? Filamentous actin is supposed to be cortical in this type of polarized epithelia. By contrast, cortical membranes are not stained. It would be great to have controls stained with CellMask or similar probe. Are there any previous studies on the SGs of Keroplatus species? Therefore, this needs control of phalloidin staining with other epithelial tissues and notably with some species of Drosophila SGs to verify that phalloidin was working properly. Presumably the authors may have a controls they could easily add to Supplemental material.

2. Pie graph in Figure 5b needs clarification: There are two large pieces - blue and red which are not mentioned or explained what they represent. Also, in both of these pieces are Orphans. There is no explanation in Figure Legend or entire text what Orphans are.

3. By reading the manuscript, one have to raise the question what are prolinins? Are these new category of previously unknown proteins? It is apparent they are, thus they need to be defined first more precisely. Most importantly, they need to be described clearly with confidence that authors "discovered" or described new category or novel type of proteins - they defined them.

4. From data (including qPCR) it is not unambiguously clear whether there exist a cocoon-unique/specific and web-unique/specific proteins. If yes, they should be picked up and listed in separate small table. Tissue and function-specific proteins (or transcripts) can shed more light on the function of Keroplatus SGs and identify their phylogenetic position reflecting adaptations to particular ecological niche.

Minor points:

1. Is it known how many larval instars Keroplatus testaceus has and what is their duration ?

2. Page 19 (Conclusion): author's statement „the cocoon silk, composed of ......provides mechanical support and protection during pupation" should be slightly modified to „protection during pupation and entire pupal stage" because cocoon stays there during whole pupal stage untill adult eclosion.

3. Authors should add arrows/arrowhead in Figure 1a and 1d to point to mucus membrane vs. fine fibers, and fibers vs. membrane sheets for easier orientation of readers that do not work with similar models.

4. Authors describe larval SG from the last instar larvae and did not mention younger larval stages. Do they have this information ?

5. In Figure 3 authors should place arrow pointing to the salivary gland or at least clearly mention that SG is a structure with green color.

6. From the Figrue 3F it is clear that salivary gland has polytene nuclei. It is a specific form of polyploidy when chromosomes stay in permanent somatic conjugation state, therefore polyteny should be mentioned in the text at least once, in order to distinguish it from typical polyploidy known in Lepidoptera.

Conclusion:

In spite of some criticism, I recommend manuscript for publication after above mentioned comments are addressed.

Reviewer #2

(Remarks to the Author)
The manuscript represents the first detailed study of the mucus web and silk structure of Keroplatus fungus gnats. The work is thorough and of high quality. It should be emphasized that fungus gnats of this genus are relatively rare and remain poorly studied. Recent studies have mainly focused on the bioluminescence mechanism.
However, the manuscript does not provide data supporting the exact species identification of the studied larvae. It is known that fungus gnats of Keroplatus testaceus and Keroplatus tipuloides are found in Europe and may be present in the same geographic areas. These species cannot be reliably distinguished at the larval stage is not possible; species identification usually requires rearing larvae to the adult stage followed by morphological analysis, or the use of DNA barcoding based on the mitochondrial COI gene.
In addition, the conclusions suggesting that the results of this study could serve as a basis for the development of hydrogels appear somewhat premature, given that the structures of individual proteins were not determined. A more cautions and nuanced formulation of these claims therefore be advisable.

Reviewer #3

(Remarks to the Author)
This study presents a solid, innovative, and multidisciplinary investigation into a novel silk system. The core findings are significant for the field of biomaterials and insect biology. However, major revisions are required to address the concerns above, particularly regarding the validation of mechanical properties under relevant conditions, functional evidence for key proteins, and quantitative structural analysis.

1.While the study acknowledges the material's high hydration-dependence, tensile tests were performed only at 30% RH. Supplementary mechanical testing under high-humidity (>90% RH) or wet-state conditions is essential, as current data may not reflect the material's native performance.

2.FTIR spectra indicate the presence of multiple secondary structures (β-sheets, α-helices, potential PPII helices). However, quantitative analysis is lacking. Fourier self-deconvolution (FSD) of the amide I band is recommended to estimate the relative proportions of different structural elements.

3.The hypothesis that peroxidases contribute to cocoon stability via dityrosine cross-linking remains speculative. Direct evidence, such as mass spectrometric detection of dityrosine or in vitro peroxidase activity assays, should be provided to support this functional claim.

4.Mechanical data are presented only for cocoon silk. The mucus silk, a key functional secretion, was not tested due to handling difficulties. Alternative approaches (e.g., microfluidic adhesion assays, nanoindentation, AFM) should be explored to evaluate its adhesive and viscoelastic properties.

5.Key parameters like Young's modulus and maximum stress are reported, but toughness (area under the stress-strain curve) is not calculated. This parameter is crucial for understanding the material's energy absorption capacity and overall performance in its natural environment.

6.The shaded areas in Fig. 4 stress-strain curves represent standard deviation, but the corresponding sample size (n) is not indicated in the figure legend or main text.

7.Please briefly justify the use of SDC and SP3 methods for proteomic sample preparation, particularly regarding their efficiency for membrane or glycoprotein extraction.

8.To confirm the presence of cross-β-sheet structures (as suggested for related glowworm silk), complementary techniques such as wide-angle X-ray scattering (WAXS) or solid-state NMR would be valuable.

9.Inconsistencies exist in journal name abbreviations within the reference list (e.g., Ref. 11, 22). A thorough check to ensure uniform formatting according to journal style is required.

Version 1:

Reviewer comments:

Reviewer #1

(Remarks to the Author)
Answers provided and changes performed by authors in their revision are satisfactory. As such I can recommend manuscript for acceptance.

Reviewer #2

(Remarks to the Author)
The authors have fully addressed my comments, therefore the MS could be recommended for acceptance.

Reviewer #3

(Remarks to the Author)
The authors have addressed my concerns; therefore, I recommend accepting this paper.

Open Access This Peer Review File is licensed under a Creative Commons Attribution 4.0 International License, which permits use, sharing, adaptation, distribution and reproduction in any medium or format, as long as you give appropriate credit to the original author(s) and the source, provide a link to the Creative Commons license, and indicate if changes were made.
In cases where reviewers are anonymous, credit should be given to 'Anonymous Referee' and the source.

The images or other third party material in this Peer Review File are included in the article's Creative Commons license, unless indicated otherwise in a credit line to the material. If material is not included in the article's Creative Commons license and your intended use is not permitted by statutory regulation or exceeds the permitted use, you will need to obtain permission directly from the copyright holder.
To view a copy of this license, visit https://creativecommons.org/licenses/by/4.0/

ANSWERS TO REVIEWERS' COMMENTS

Reviewer #1

1. Phalloidin which is supposed to bind filamentous actin in Figure 3F is red color, but red signal is diffused over whole cytoplasm. Is this real? Filamentous actin is supposed to be cortical in this type of polarized epithelia. By contrast, cortical membranes are not stained. It would be great to have controls stained with CellMask or similar probe. Are there any previous studies on the SGs of Keroplatus species? Therefore, this needs control of phalloidin staining with other epithelial tissues and notably with some species of Drosophila SGs to verify that phalloidin was working properly. Presumably the authors may have a controls they could easily add to Supplemental material.

Response: Thank you for your comment. The diffuse red signal arises from the confocal z-stack projection of multiple optical sections, which causes the actin cortex to appear blurred. To clarify this, we have included a control image (S1) showing a single optical section in which the actin cortex is clearly focused. This control image has been added to the supplementary materials.

2. Pie graph in Figure 5b needs clarification: There are two large pieces - blue and red which are not mentioned or explained what they represent. Also, in both of these pieces are Orphans. There is no explanation in Figure Legend or entire text what Orphans are.

Response: Thank you for your comment. The term orphans denotes proteins that have no known close homologs. An explanation of the term "orphans" has been added to the legend for Figure 5.

3. By reading the manuscript, one have to raise the question what are prolinins? Are these new category of previously unknown proteins? It is apparent they are, thus they need to be defined first more precisely. Most importantly, they need to be described clearly with confidence that authors "discovered" or described new category or novel type of proteins - they defined them.

Response: Thank you for your comment. We added following explanation to page 11 (lines 272–274). These Plins represent proline-rich secreted proteins containing 10–18% proline, lacking recognizable conserved domains or detectable homologs outside this species, and predicted to be largely disordered (Tab. S2B).

4. From data (including qPCR) it is not unambiguously clear whether there exist a cocoon-unique/specific and web-unique/specific proteins. If yes, they should be picked up and listed in separate small table. Tissue and function-specific proteins (or transcripts) can shed more light on the function of Keroplatus SGs and identify their phylogenetic position reflecting adaptations to particular ecological niche.

Response: Thank you for your comment. Yes, as clarified on page 9 (lines 213–214) for the proteomic analysis, no proteins were detected exclusively in the cocoon or in either material across biological replicates. and on page 12 (lines 290–291). Similarly, for the qPCR we observed 1 or 2 orders of magnitude difference in abundance between salivary glands and control tissues. - none of the analyzed transcripts were found to be exclusively expressed in an SG. However, their expression levels differed by one to two orders of magnitude compared with the control tissues.

Upon reanalysis using a fully reproducible workflow in R, we confirmed significant overall differences among tissues using the Kruskal–Wallis test. However, pairwise comparisons did not remain statistically significant after correction for multiple testing.

To ensure strict statistical rigor and reproducibility, we have removed the letter annotations from the figures and now report only the overall test results. The figures and statistical description have been updated accordingly.

5. Is it known how many larval instars Keroplatus testaceus has and what is their duration?

Response: Thank you for your comment. The number of larval instars and their duration are not documented for *Keroplatus testaceus*. Fang et al. (2018) Entomologie heute 30) observed four larval instars in related species *Keroplatus nipponicus*.

6. Page 19 (Conclusion): author's statement „the cocoon silk, composed of ......provides mechanical support and protection during pupation" should be slightly modified to „protection during pupation and entire pupal stage" because cocoon stays there during whole pupal stage untill adult eclosion.

Response: Thank you for your comment. The statement has been modified on page 19 (line 478).

7. Authors should add arrows/arrowhead in Figure 1a and 1d to point to mucus membrane vs. fine fibers, and fibers vs. membrane sheets for easier orientation of readers that do not work with similar models.

Response: Thank you for your comment. Arrows and arrowheads have been added to Figures 1A, 1B, and 1D, and the corresponding explanations have been included in the Figure 1 caption (page 5).

8. Authors describe larval SG from the last instar larvae and did not mention younger larval stages. Do they have this information ?

Response: Thank you for your comment. Since the *K. testaceus* larvae are difficult to maintain under laboratory conditions, we focused on last-instar larvae and specifically selected individuals measuring approximately 15–20 mm. Histological sections were prepared from these individuals. Subsequent pupation of some individuals in the laboratory confirmed that they represented the final larval stage of *K. testaceus.*

9. In Figure 3 authors should place arrow pointing to the salivary gland or at least clearly mention that SG is a structure with green color.

Response: Thank you for your comment. As suggested, it has been clearly stated that SG is a structure with green color in Figure 3 caption (page6).

10. From the Figrue 3F it is clear that salivary gland has polytene nuclei. It is a specific form of polyploidy when chromosomes stay in permanent somatic conjugation state, therefore polyteny should be mentioned in the text at least once, in order to distinguish it from typical polyploidy known in Lepidoptera.

Response: Thank you for your comment. We have added it on page 7 (lines 162-164) as suggested.

Reviewer #2

1) The manuscript does not provide data supporting the exact species identification of the studied larvae. It is known that fungus gnats of Keroplatus testaceus and Keroplatus tipuloides are found in Europe and may be present in the same geographic areas. These species cannot be reliably distinguished at the larval stage is not possible; species identification usually requires rearing larvae to the adult stage followed by morphological analysis, or the use of DNA barcoding based on the mitochondrial COI gene.

Response: Thank you for your comment. Indeed, we performed barcoding verification of our Keroplatus testaceus larvae. The sequence of COI has been added to the Supplementary Data (Fig. S5).

2) In addition, the conclusions suggesting that the results of this study could serve as a basis for the development of hydrogels appear somewhat premature, given that the structures of individual proteins were not determined. A more cautions and nuanced formulation of these claims therefore be advisable.

Response: Thank you for your comment. The statements regarding hydrogels have been revised on page 19 (lines 479–480).

Reviewer #3

1.While the study acknowledges the material's high hydration-dependence, tensile tests were performed only at 30% RH. Supplementary mechanical testing under high-humidity (>90% RH) or wet-state conditions is essential, as current data may not reflect the material's native performance.

Response: Thank you for your comment. We fully agree that, given the pronounced hydration sensitivity of this system, mechanical characterization under high-humidity or fully hydrated conditions is highly relevant for evaluating its native performance. We acknowledge that the mechanical data presented therefore reflect the material's behavior under controlled dry-to-moderate humidity conditions rather than fully hydrated native conditions. To address your concern, we have revised the manuscript to explicitly state this limitation in the Methods and Discussion sections. We avoided overgeneralizing the mechanical performance to fully hydrated environments. We added a statement highlighting high-humidity and wet-state mechanical characterization as an important direction for future investigation.

2.FTIR spectra indicate the presence of multiple secondary structures (β-sheets, α-helices, potential PPII helices). However, quantitative analysis is lacking. Fourier self-deconvolution (FSD) of the amide I band is recommended to estimate the relative proportions of different structural elements.

Response: Thank you for your comment. The FSD analyses were included in supporting information (Figure S7 and S8) as well as discussion and experimental parts were updated accordingly

3. The hypothesis that peroxidases contribute to cocoon stability via dityrosine cross-linking remains speculative. Direct evidence, such as mass spectrometric detection of dityrosine or in vitro peroxidase activity assays, should be provided to support this functional claim.

Response: We thank the reviewer for this important and constructive comment and agree that direct biochemical evidence, such as mass spectrometric identification of dityrosine cross-links or in vitro peroxidase activity assays, would provide definitive support for the proposed mechanism.
In the present study, our interpretation is based on indirect evidence, including [e.g., the presence of peroxidase transcripts/proteins, oxidative environment, tyrosine-rich protein sequences, structural stability of the cocoon, etc.], which are consistent with previously reported peroxidase-mediated dityrosine cross-linking mechanisms in structural protein systems. However, we acknowledge that this does not constitute direct confirmation of dityrosine formation.
We note that dityrosine cross-links involve stable C–C bonds that are not reducible (in contrast to disulfide bonds), and their detection in complex biological matrices requires specialized cross-linking mass spectrometry workflows and dedicated analysis software. Such analyses are typically performed on purified proteins or simplified in vitro systems. Due to the compositional complexity of the cocoon matrix and the absence of targeted cross-linking MS infrastructure within the scope of this study, direct detection of cross-linked peptides was not feasible.

To address your concern, we have revised the manuscript and removed definitive language implying direct mechanistic proof. We clearly state that the proposed role of peroxidases in mediating dityrosine cross-linking remains a hypothesis. We explicitly identify mass spectrometric cross-link mapping and in vitro enzymatic assays as important directions for future investigation.
We believe this revision appropriately aligns our conclusions with the level of evidence currently available and prevents overinterpretation of the data. We appreciate the reviewer's suggestion, which has helped us refine and clarify the mechanistic scope of our study.

4. Mechanical data are presented only for cocoon silk. The mucus silk, a key functional secretion, was not tested due to handling difficulties. Alternative approaches (e.g., microfluidic adhesion assays, nanoindentation, AFM) should be explored to evaluate its adhesive and viscoelastic properties.

Response: Thank you for your comment. We fully agree that techniques such as microfluidic adhesion assays, nanoindentation, or AFM-based rheological measurements would provide important quantitative insights into the adhesive and viscoelastic properties of the mucus secretion. However, due to the extremely small volume, rapid curing dynamics, and handling instability of freshly secreted mucus, reliable isolation and mechanical testing were not technically feasible within the scope of the present study. To partially address this concern, we have added Supplementary Video S9 which documents real-time mucus secretion and cocoon construction behavior. The video illustrates the immediate adhesive interaction of mucus with the substrate and its extensibility during larval manipulation,

5. Key parameters like Young's modulus and maximum stress are reported, but toughness (area under the stress-strain curve) is not calculated. This parameter is crucial for understanding the material's energy absorption capacity and overall performance in its natural environment.

Response: Thank you for your comment. We added the data on *toughness* to supplemetary data Tab. S1.

6.The shaded areas in Fig. 4 stress-strain curves represent standard deviation, but the corresponding sample size (n) is not indicated in the figure legend or main text.

Response: Thank you for your comment. We added the sample size to the caption of Figure 4 (page 8).

7.Please briefly justify the use of SDC and SP3 methods for proteomic sample preparation, particularly regarding their efficiency for membrane or glycoprotein extraction.

Response: Thank you for your comment. SDC disrupts lipid bilayers and efficiently solubilizes challenging proteins while remaining compatible with downstream mass spectrometry. It is therefore particularly effective for membrane proteins and glycoproteins. SP3 enables efficient capture and cleanup of hydrophobic and glycosylated proteins, minimizing losses during washing. It also improves digestion efficiency and is well suited for membrane- and glycoprotein-focused workflows where careful sample handling is critical. Together, SDC provides strong solubilization of difficult proteins, while SP3 ensures high recovery and clean preparation, making both methods well justified for proteomic analyses focused on membrane proteins and glycoproteins.

8. To confirm the presence of cross-β-sheet structures (as suggested for related glowworm silk), complementary techniques such as wide-angle X-ray scattering (WAXS) or solid-state NMR would be valuable.

Response: Thank you for this insightful suggestion. We agree that complementary structural techniques such as wide-angle X-ray scattering (WAXS) or solid-state NMR would provide powerful and direct evidence for the presence of cross-β-sheet structures in the cocoon silk.
In the present study, our interpretation of potential cross-β-sheet organization was based on indirect evidence, including e.g., FTIR amide I band analysis. Due to limitations in sample quantity and access to synchrotron/WAXS or solid-state NMR facilities during the course of this study, we were unable to perform these additional structural analyses. To address the reviewer's concern, we have revised the manuscript to moderate our structural claims and avoid definitive statements regarding cross-β-sheet architecture. We clearly state that the presence of cross-β-sheet structures is inferred but not directly confirmed. We add a statement in the Discussion highlighting WAXS and solid-state NMR as important future approaches to conclusively resolve the secondary structure organization.

9. Inconsistencies exist in journal name abbreviations within the reference list (e.g., Ref. 11, 22). A thorough check to ensure uniform formatting according to the journal style is required.

Response: Thank you for your comment. References have been edited.
