# Supplementary material for: Characterization of silk-like materials from larval salivary glands of the dipteran, Keroplatus testaceus
Source: Nat Commun. 2026 Oct 5;17:11363. doi: 10.1038/s41467-026-78291-5 (PMC13728221; doi:10.1038/s41467-026-78291-5)
Supplement: Supplementary file 5 [file 41467_2026_78291_MOESM3_ESM.docx]

| **Protein name** | **Gene symbol** | **FW** | **RV** | **PS (bp)** | **Tm (°C)** |
| --- | --- | --- | --- | --- | --- |
| Collagen-like | *Kt_Coll-L* | gcagcatcaagtagtgcagc | acccggtgatggtgatgttc | 129 | 60 |
| Sericin-like | *Kt_Ser-L* | actcctcgtcggcattgttg | tgctgaaacaccaccactga | 172 | 60 |
| Prolinin 1 | *Kt_Plin1* | tggacctgtatcgttggcaa | cacttgctttccaccactgc | 178 | 60 |
| Prolinin 6 | *Kt_Plin6* | cagctccaacttctccagca | gaaggcgttgcaggagtact | 157 | 60 |
| von Willebrand factor 1 | *Kt_vWC1* | caatcgggtgcgcttgtaac | tctcggcctttgctaaccaa | 104 | 60 |
| Zonadhesin-like 1 | *Kt_Zon-L1* | tacttgttgggctgaggctg | ttaacgggtgctatcggtcg | 144 | 60 |
| Serpin 1 | *Kt_Srp1* | ccaagccagccaacaattcc | caggagtcgatggtacgctg | 163 | 60 |
| Serpin 2 | *Kt_Srp2* | gtatctaccaccgtcgccag | ctggttctcctgcttgtggt | 143 | 60 |
| Prolinin 8 | *Kt_Plin8* | cagacttgcccggtgttaca | aacgtgatcggcttcactgt | 116 | 60 |
| AL rich protein 3 | *Kt_ALRP3* | tgctctcagttagcagcacc | tgcattttctgttcggcagc | 106 | 60 |
| A rich protein 1B | *Kt_ARP1B* | acaacagacattgggagcga | gccgcttttatgcccgtaac | 147 | 60 |
| KPDA tail protein | *Kt_KPDA* | actggtccatctgctgatgc | gatggtgttggcgttgcatt | 185 | 60 |
| SL rich protein | *Kt_SLRP* | gcgtgaatactctcccagca | ggtggtgaaccgctcaaaac | 135 | 60 |
| CW protein 2A | *Kt_CWP2A* | atggctgggtggtgttgaat | ttcatcgaaaaatcaaccacgt | 152 | 60 |
| YL rich protein | *Kt_YLRP* | accggccagtaaattgccat | gtggattaccgcccaacgta | 164 | 60 |
| Chitin-binding protein 1 | *Kt_ChitBP1* | tgtgtactggttgcggaagg | cactgtgcatttggtctggc | 151 | 60 |

**Supplementary Data 1.**
