# Supplementary material for: Characterization of silk-like materials from larval salivary glands of the dipteran, Keroplatus testaceus
Source: Nat Commun. 2026 Oct 5;17:11363. doi: 10.1038/s41467-026-78291-5 (PMC13728221; doi:10.1038/s41467-026-78291-5)
Supplement: Supplementary file 6 [file 41467_2026_78291_MOESM4_ESM.docx]

Kt_Prolinin 4

Kt_Prolinin 3

Kt_Prolinin 1

Kt_Prolinin 2

Kt_Prolinin 8

Kt_Prolinin 7

Kt_Prolinin 6

Kt_Prolinin 5

Kt_Serpin3B

Kt_Serpin 3A

Kt_Serpin 2

Kt_Serpin 1

Kt_Serpin 6

Kt_Serpin5

Kt_Serpin 4

Kt_Serpin 7

Kt_Chitin-binding protein 1

Kt_Chitin-binding protein 2

Kt_Chitin-binding protein 3

Kt_Sericin-like

Kt_Collagen-like

Kt_Chitin-binding protein 4

Kt_KPDA tail protein

Kt_PK rich protein

Kt_EK_rich_protein

Kt_EQA rich protein

Kt_Mucin-like1

Kt_Mucin-like2

Kt_IA rich protein

Kt_CW protein 2A

Kt_CW protein 3

Kt_Isomerase

Kt_Metalloaminopeptidase 2

Kt_SL rich protein

Kt_TP rich protein

Kt_L rich protein 2

Kt_Zonadhesin-like 1

Kt_Kazal 1A

Kt_LN rich protein 11 kDa

Kt_L rich protein 1
